# Neoadjuvant PD-L1/TGF-**β** blockade can rapidly disrupt the tumor stroma in head and neck cancer patients

**DOI:** 10.1172/JCI198718

**Published:** 2026-11-02

**Authors:** Caroline Hoffmann, Jan-Timon Werle, Philémon Sirven, Mamy Andrianteranagna, Jocelyn Gal, Wiem Lassoued, Jerzy Klijanienko, Jeremy Mesple, Benjamin Demaille, Abdenour Abbas, Valentine Gauthier, Kyra J.E. Borgman, Isabelle Puisieux, Serena Janho dit Hreich, Olivier Choussy, Gregoire Marret, Sonia Lameiras, Nathalie Badois, Antoine Dubray-Vautrin, Gilles Dolivet, Lionnel Geoffrois, Renaud Leclère, Wail Zeitouni, Noemie Robil, Jane Merlevede, Altynbek Zhubanchaliyev, Maud Kamal, Ivan Bieche, Laure Monard, Clotilde Simon, Delphine Louis, Clint T. Allen, Arun Rajan, James L. Gulley, Maude Guillot-Delost, Christophe Le Tourneau, Hélène Salmon

**Affiliations:** 1 Equipe Leader Fondation ARC, INSERM U932, Immunity and Cancer, Institut Curie, PSL University, Paris, France.; 2 Surgical Oncology Department, Institut Curie, Paris, France.; 3 Department of Biostatistics, Centre Lacassagne, Nice, France.; 4 Center for Immuno-Oncology, Center for Cancer Research, National Cancer Institute, National Institutes of Health, Bethesda, Maryland, USA.; 5 Department of Pathology and; 6 Single Cell Initiative, Institut Curie, Paris, France.; 7 Department of Drug Development and Innovation (D3i), Institut Curie, Paris & Saint-Cloud, INSERM U900 Research Unit, Paris-Saclay University, Orsay, France.; 8 Institut de Cancérologie de Lorraine, Vandœuvre-lès-Nancy, France.; 9 GenoSplice, Paris, France.; 10 Inserm U1331, Institut Curie, PSL University, Paris, France.; 11 Institut Hospitalo Universitaire PRISM, National PRecISion Medicine Center in Oncology, Gustave Roussy, Paris, France.; 12 Department of Genetics, Institut Curie, Paris, France.; 13 Unicancer, Le Kremlin-Bicêtre, France.; 14 Section on Translational Tumor Immunology, National Institute on Deafness and Other Communication Disorders, and; 15 Thoracic and Gastrointestinal Malignancies Branch, Center for Cancer Research, National Cancer Institute, National Institutes of Health, Bethesda, Maryland, USA.

**Keywords:** Immunology, Oncology, Cancer immunotherapy, Clinical trials, Head and neck cancer

## Abstract

**BACKGROUND:**

TGF-β drives fibroblast activation in cancer and is a resistance biomarker to PD-1/PD-L1 blockade.

**METHODS:**

Focusing on the tumor stroma, we characterized the impact of neoadjuvant treatment with bintrafusp alfa (BA), a dual PD-L1 and TGF-β inhibitor, in a window-of-opportunity study with 6 patients with head and neck squamous cell carcinoma (HNSCC). Tumor biopsies before treatment and 3 weeks after therapy were analyzed using scRNA-seq, imaging, and spatial transcriptomics.

**RESULTS:**

After treatment, we observed significantly decreased levels of activated LRRC15^+^ cancer-associated fibroblasts (CAFs), remodeled stromal architecture with reduced collagen I and XII content, and increased CD8^+^ T cell infiltration in immune-excluded tumors. These changes were independently confirmed in an additional neoadjuvant BA-treated cohort of 6 patients with HNSCC. The drastic CAF and stromal compartment reduction observed indicated a fibroblast depletion rather than a fibroblast polarization shift.

**CONCLUSION:**

While our final clinical cohort does not allow assessment of the treatment effect, this study demonstrated that targeting TGF-β in patients can disrupt the tumor stroma within a short time frame. Further trials and novel biomarkers are now needed to select patients who could benefit.

**TRIAL REGISTRATION:**

ClinicalTrials.gov NCT04428047.

**FUNDING:**

Merck KGaA, ARC Emerging Leaders in Oncology, ANR-10-IDEX-0001-02 PSL, Centre d’Investigation Clinique IGR-Curie 1428, Site de Recherche Intégrée sur le Cancer (INCa-DGOS-Inserm-ITMO cancer_18000), French Government “Investissements d’Avenir” program, France 2030 program, Fondation pour la Recherche Médicale, Marie Skłodowska-Curie grant agreement 847718, Cooperative Research and Development Agreement with EMD Serono, and NIH (ZIA BC 010666).

## Abstract 2

This study shows that neoadjuvant treatment with TGFβ/PD-L1 blockade can eliminate activated fibroblasts and increase T cell infiltration in immune-excluded tumors of HNSCC patients.

## Introduction

In recent decades, cancer immunotherapies have succeeded in increasing the specificity and functionality of both adoptively transferred and endogenous T cells. Nonetheless, their efficacy in treating solid tumors remains limited (1–3). Cytotoxic T cells need to contact target cells to unleash their full potential, yet various components of the tumor microenvironment (TME) often contribute to their exclusion, creating obstacles for immunotherapy efficacy. Previous work by us and others has shown that a dense ECM can hinder T cell infiltration and motility (4, 5) and that tumor cell islets are often surrounded by multiple layers of ECM-producing cancer-associated fibroblasts (CAFs) in advanced solid tumors (6). Various preclinical studies, such as FAP-targeted CAR T cell–mediated depletion (7–9) or LRRC15^DTR^ depletion (10), have provided proof of concept that stromal cell targeting can enhance endogenous, vaccine-stimulated, and adoptively transferred CAR CD8^+^ T cell–based antitumor immunity. However, little is known about the biological impact of targeting the stroma in patients with cancer.

TGF-β has historically been a target of interest due to its pivotal roles in cancer progression, including promoting an immune-evasive TME and desmoplasia and contributing to tumor cell plasticity, dissemination, and resistance (11). Initial trials with single-agent TGF-β inhibitors produced disappointing results and adverse systemic effects (12, 13); however, the potential to combine them with immunotherapy has led to renewed interest and drug development. Indeed, a TGF-β signaling signature in fibroblasts has been associated with poorer response to PD-L1 blockade in patients with advanced urothelial cancer, specifically for those with immune-excluded tumors (14). This signature was further refined specifically to TGF-β–driven LRRC15^+^ CAFs, which have been detected in multiple human cancer types, including pancreatic, breast, lung, and head and neck cancers (15).

These findings establish a clear rationale for targeting TGF-β in combination with immunotherapy. One promising candidate is bintrafusp alfa (BA), a fusion protein that blocks both PD-L1 and TGF-β. Functionally, its anti–PD-L1 (aPD-L1) moiety targets BA to the tumor, and its TGF-β blocking effect is expected to more specifically target the TME than broader TGF-β–targeting drugs (16). In multiple preclinical models, BA demonstrated superior antitumor activity than either aPD-L1 alone or combined with anti–TGF-βRII (17, 18). Mechanistic studies in vitro and in vivo, in preclinical animal models, revealed several potential modes of action for BA, including dampening the immunosuppressive ability of Tregs, decreasing fibroblast activation and fiber deposition, and activating CD8^+^ T cells and NK cells in both the periphery and the tumor (17, 19–21).

BA demonstrated promising results in a phase I trial in head and neck squamous cell carcinoma (HNSCC), with an objective response rate of 27.9% for the overall cohort population, 13.6% for patients who were HPV^–^, and 50.0% for those who were HPV^+^ (ClinicalTrials.gov NCT02517398) (22). A neoadjuvant preoperative study in patients with HPV^–^ HNSCC reported a mean pathological response (PathR) rate of 36% (23). Nonetheless, BA faced setbacks in clinical trials, and both the phase II/III trial in biliary tract cancer (INTR@PID BTC 055; ClinicalTrials.gov NCT04066491) and the phase III trial in non–small cell lung cancer (INTR@PID Lung 037; ClinicalTrials.gov NCT03631706) were discontinued due to the unlikely achievement of their primary objective of improving outcomes compared with standard care, despite encouraging preexisting phase II results. However, stroma targeting strategies are still widely investigated, and the biological effects on the mesenchymal stroma in tumor patients are not known.

Here, we report the findings of a window-of-opportunity study within the framework of a phase II trial (ClinicalTrials.gov NCT04428047) we initiated, testing short neoadjuvant preoperative administration of 2 doses of BA in patients with untreated, resectable HPV^–^ HNSCC. To determine whether BA induced biological changes in the TME, we harnessed 6 paired biopsy samples before and 3 weeks after treatment. We used combined scRNA-seq and multiplexed imaging on the 6 sets of tumor samples. BA treatment reduced TGF-β signaling, with the strongest impact on mesenchymal cells. We also observed a strong remodeling of the stromal architecture, with a systematic decrease in LRRC15^+^ activated CAFs and an increase in CD8^+^ T cell infiltration in immune-excluded samples with a CAF capsule. The strongest PathR was observed for a patient with an initially T cell–excluded tumor but containing lymphoid aggregates. Notably, tumor resection analysis of this responder patient, in addition to biopsies, highlighted a spatial heterogeneity of the treatment response, especially with maintenance of LRRC15^+^ CAFs around stemness-associated tumor cells. Confirmed in an independent BA-treated cohort of 6 additional patients with HNSCC (ClinicalTrials.gov NCT04247282) (23), our results show the rapid and drastic effects of PD-L1/TGF-β blockade on stroma remodeling in patients with cancer.

## Results

### Patient characteristics and clinical outcomes.

Patients included in this study were enrolled in a single-arm phase II clinical trial (NCT04428047) assessing neoadjuvant administration of 2 doses of 1,200 mg of BA at a 2-week interval, prior to surgery after 3 weeks (Figure 1). The 6 patients who received the 2 doses were primarily untreated HPV^–^ HNSCC patients of the oral cavity (*n* = 4) and the oropharynx (*n* = 2) and staged T2N1–3 and T3 at any N at M0 (Figure 2A). Although 1 tumor (patient Pt07) stained positive for p16, a protein often used as a surrogate marker for HPV^+^ carcinoma, all patients were confirmed to be HPV^–^ by touchdown general primer (GP6^+^) PCR.

Pre- (PRE) and posttreatment (POST) biopsies were collected by endoscopy in a similar manner to optimize comparability for biological responses. As defined in the clinical trial, the treatment efficacy endpoint was assessed by PathR on the tumor resection. Patients with specimens showing tumor cell necrosis and/or giant cell/histolytic reaction to keratinous debris in ≥ 10% and ≥ 50% of tumor area were considered partial responders (PRs) and major partial responders (MPRs), respectively. These thresholds are adapted to such short neoadjuvant regimens, with the 10% threshold selected based on a phase II trial evaluating short neoadjuvant pembrolizumab in HNSCC (24). Three patients (Pt02, Pt04, and Pt05) were PRs (3/6 patients, 50%), and 1 (Pt07) was MPR, with PathR = 54% (1/6 patients, 17%). Two patients (Pt03 and Pt08) were nonresponders (2/6 patients, 33%). Detailed clinical characteristics are summarized in Figure 2A, Supplemental Figure 1A, and Supplemental Table 1, and representative hematoxylin, eosin, and saffron images across the spectrum of patient responses are in Supplemental Figure 1B (supplemental material available online with this article; https://doi.org/10.1172/JCI198718DS1). We next evaluated the association between PD-L1 expression on tumor or immune cells and PathR (Figure 2B, Supplemental Figure 1C, and Supplemental Table 2). PD-L1 staining in PRE tumor biopsies showed a combined positive score (defined as number of PD-L1^+^ cells/number of viable tumor cells) ranging from 0 to 30, which showed no positive correlation with PathR (Spearman’s *r* = 0.20, *P* = 0.714). When considering immune cells only (immune positive score, number of PD-L1^+^ immune cells/number of viable tumor cells), ratios ranged from 0 to 13 and were positively correlated with PathR (Spearman’s *r* = 0.90, *P* = 0.028).

To ensure we were capturing clear biological features associated with response, we focused on the case with a very distinct response (MPR case, Pt07, >50% PathR). Next-generation sequencing (NGS) showed that the major responder had the most detected oncogene alterations and the highest tumor mutational burden (TMB) (Figure 2C), although all patients were still classified as “TMB low” relative to a previously established dataset (25). We identified deleterious mutations of NSD1, RB1, and CDKN2A (p16) in this patient that explained the accumulation of p16 in cancer cells in the absence of HPV (Figure 2A) (26). Another specificity of the Pt07 PRE biopsy was a higher number of immune aggregates in the stroma, composed of T cells, B cells, and DC-LAMP^+^ DCs and surrounded by plasma cells (Figure 2D), as detected by multiplex IHC. Notably, the stromal densities of these immune cells were strongly increased in this patient after BA treatment (Supplemental Figure 1D). Pt07 CD68^+^ macrophages also massively infiltrated the stroma after treatment, especially the zones surrounding necrotic tumor cells (Supplemental Figure 1E). Together with the high PD-L1 immune positive score observed for Pt07, these results could suggest a higher antitumor immunity potential prior to treatment for the MPRs, likely unleashed by the PD-L1/TGF-β inhibition.

### BA treatment reduces TGF-β signaling, with the strongest impact on mesenchymal cells.

To dissect the biological impact of BA treatment on cancer cells and the TME, we analyzed the 6 paired biopsies by multiplex imaging and 5 pairs by scRNA-seq (1 POST scRNA-seq run [Pt05] failed). scRNA-seq analysis identified 13 clusters of major cell types (see Methods; Figure 3A, Supplemental Figure 2A, and Supplemental Table 3); all major cell types were well represented in all baseline samples (Supplemental Figure 2B). To identify broad changes induced by BA administration, irrespective of response or patient specificity, we first performed a global PRE/POST comparison by performing pseudobulk analysis by patient, cell type, and PRE or POST status and used DESeq2 to compute differentially expressed genes (DEGs) for each patient and cell type (Figure 3, B and C). Overall, we found 60 significantly upregulated DEGs and 96 significantly downregulated DEGs in the POST samples compared with the PRE samples [adjusted *P* < 0.05; |log_2_(fold-change)| > 0.5; Figure 3B, Supplemental Figure 3, and Supplemental Table 4]. The top 20 upregulated and downregulated genes are depicted in Figure 3B. Upregulated genes included multiple chemokines (*CXCL8*, *CXCL9*, and *CXCL10*), as well as *IFNG*, which encodes IFN-γ. The downregulated genes were enriched for TGF-β–inducible genes, with multiple matrix-associated components (e.g., *COL1A1, COL3A1*, *COL11A1*, *MMP2*, *POSTN*, and *ASPN*) (Figure 3B, Supplemental Figure 3, and Supplemental Table 4). DEGs were analyzed within each cluster to investigate their cellular origin and to identify the cell types most affected by BA treatment (Figure 3C and Supplemental Table 4). Figure 3C shows the top DEGs from Figure 3B within each cell type before and after treatment (log average expression). As expected, *IFNG* was mainly expressed and upregulated in CD8^+^ T cells and in NK cells. All cell types showed an upregulation of IFN-γ–inducible genes (e.g., *CXCL9*, *IDO1*, *MMP12*, *WARS*, and *GBP1*), with monocytes/macrophages and DCs exhibiting the strongest signature of IFN-γ activation (Figure 3C). The downregulated matrix-related genes mostly came from mesenchymal cells, especially CAFs and pericytes (Figure 3C), in line with their higher expression levels of TGF-β coreceptors (Supplemental Figure 2, C and D). These results confirmed that TGF-β signaling was reduced in the TME after BA treatment and that mostly stromal cells were affected.

To further investigate whether the decrease in TGF-β signaling could be attributable to the aPD-L1 moiety of BA, we performed the same analysis with a publicly available scRNA-seq dataset from patients with HNSCC who had been treated only with aPD-L1 durvalumab (Supplemental Figure 4) (27). In aPD-L1–treated patient data, we found a similar increase of IFN-γ–activated monocytes/macrophages and DCs with upregulated *CXCL9* and *IDO1*, yet no decreased expression of collagens or other TGF-β target genes was observed (Supplemental Figure 4, A and B). Taken together, these results highlight the antifibrotic effect of blocking TGF-β signaling, which was recapitulated in the BA-treated patients in our study.

### BA treatment induces a strong remodeling of the stromal architecture.

Given the strong TGF-β blocking effect observed on mesenchymal cells, we sought to investigate changes in the stromal architecture. Masson’s trichrome staining revealed drastic posttreatment modifications of global collagen fibers (Figure 4A); we therefore thought to stain and quantify specific types of collagens in all PRE and POST samples. This included the major matrix component collagen I, as well as collagen XII, recently identified as part of a fibrillar barrier associated with T cell exclusion in human lung cancer (6) and collagen IV coverage (Figure 4, B and C). We found reduced collagen I (*P* = 0.062) and collagen XII (*P* = 0.062) in 5 of 6 patients (Figure 4, B and C). In contrast, collagen IV, which is more specifically expressed by pericytes and endothelial cells (Supplemental Figure 2A), showed no global reduction (*P* = 0.844; Figure 4C).

We noticed not only a strong reduction in fibers but also a deep impact on the stroma, with changes in its proportion within the tumor lesion. By applying a stroma and tumor classifier on each PRE and POST sample, we observed a markedly reduced stromal area in a subset of patients, especially Pt02 and Pt04 (Figure 4D). To further investigate the cellular changes underlying this stromal loss, we additionally performed spatial transcriptomic analysis (MERSCOPE technology; see Methods) on the paired PRE and POST samples for Pt04 (Figure 4E). Based on literature showing CAFs as major ECM producers in the TME, and our results showing strong transcriptomic changes in CAFs after BA treatment (Figure 3C), we hypothesized that fibroblasts would be the main impacted cell population. The gene panel was designed to distinguish the main cell types (see Methods; Supplemental Table 8), allowing their quantification after segmentation and clustering. This analysis confirmed the strong decrease in CAFs after BA treatment in Pt04, without reduction in endothelial cell density (Figure 4E).

Given that Pt08 behaved differently with an increased proportion of stroma in the whole lesion (Figure 4D, right), we performed the same spatial transcriptomic analysis on Pt08 PRE and POST samples. However, the MERSCOPE analysis revealed a 4-fold reduction in CAFs after BA treatment (Supplemental Figure 5A). The increased stromal area might be explained by a massive infiltration of immune cells observed in the POST biopsy for this patient (Supplemental Figure 5B), who exhibited bleeding after treatment (Supplemental Table 14).

To our knowledge, our results are the first demonstration that TGF-β– and stromal-oriented drugs can have a rapid and drastic impact on fibroblasts and tumor architecture in cancer patients.

### BA induces a systematic decrease in LRRC15^+^ activated CAFs.

To investigate whether several subtypes/states of fibroblasts in the PRE lesions were differentially impacted by the treatment, we further analyzed the scRNA-seq dataset from all patients. Subclustering of the CAFs and pericytes revealed 1 clearly distinct pericyte cluster (expressing *MCAM*, *RGS5*, and *COX4I2*) and 2 CAF subsets (Figure 5A and Supplemental Table 5). We observed 1 CAF subcluster, denoted CD10^+^/IL24^+^ CAF, that expressed *MME* (CD10) as well as genes encoding various cytokines and chemokines (*IL24*, *CXCL1*, *IL6*, and *CXCL6*), resembling inflammatory CAFs (iCAFs) described across multiple cancer types (28, 29). The second fibroblast cluster, denoted LRRC15^+^ CAF, expressed a similar transcriptional profile as myofibroblastic CAFs (myCAFs) (15, 28), with the activation marker *LRRC15* and ECM-related genes (*CTHRC1*, *COL11A1*, and *MMP11*). Proportion analysis within the total CAF clusters showed a global reduction in the LRRC15^+^ CAFs (Figure 5B). To confirm this, we quantified LRRC15^+^ and CD10^+^ CAF abundance through coverage of the stroma in PRE and POST samples (Figure 5, C and D). This showed a significant (*P* = 0.031, paired Wilcoxon’s test) reduction of the LRRC15^+^ myCAFs in all 6 patients, while CD10^+^ iCAF coverage (quantified as CD10^+^CD45^–^) of the stroma was not significantly modified (Figure 5D).

We then performed additional multiplex IHC analyses incorporating markers of apoptosis (cleaved caspase-3) and cell cycle arrest/senescence (p21, p16, and γH2AX). These analyses revealed the presence of cleaved caspase-3^+^LRRC15^+^ CAFs in POST biopsies (Supplemental Figure 5C), consistent with ongoing apoptosis. CAFs did not express γH2AX or p16, yet a fraction of residual LRRC15^+^ CAFs expressed p21 (Supplemental Figure 5D). p21 staining was absent in CAFs prior to treatment, suggesting some phenotypic modulation among the fibroblasts that persist after therapy. When considered together with the overall reduction in CAF abundance (Figure 4E, Figure 5, C and D, and Supplemental Figure 5A), these findings support a model in which treatment induces predominant depletion of LRRC15^+^ CAFs, along with some phenotypic modulation in residual cells.

To investigate the stromal loss and activated CAF changes in a validation cohort, immunofluorescence (IF) staining was performed on 6 PRE and POST samples from an NIH patient cohort treated with a similar regimen (window-of-opportunity trial, preoperative D1-D14 BA injections in patients with HPV^–^ HNSCC) (23). This confirmed the significant (*P* = 0.031, paired Wilcoxon’s test) decrease in LRRC15^+^ CAFs for all 6 additional patients (Supplemental Figure 6, A and B) as well as a marked reduction in the whole stromal compartment in 4 of 6 patients (Supplemental Figure 6, C and D).

Altogether, these results obtained in 2 independent cohorts point toward a dominant CAF depleting effect of BA rather than a simple fibroblast polarization shift toward a less activated CAF state.

### BA treatment induces changes in CD4^+^ and CD8^+^ T cell infiltration.

We previously showed in human lung carcinoma tumors that activated CAFs can form a dense fibrillar capsule around tumor islets, associated with CD3^+^ and CD8^+^ T cell marginalization in the stroma (6). Given the stromal disruption observed (Figure 4), we analyzed T cell subset infiltration after BA treatment. While we found an overall reduction in T cell whole-lesion density, especially for conventional (significant decrease) and Treg CD4^+^ T cell density (Figure 5E, top row), T cell distribution in tumor and stroma was changed (Figure 5E, middle and bottom rows, and Figure 5F). We observed an increased tumor/stroma CD8^+^ T cell density ratio in 5 patients out of 6. Analyzing CAF topological arrangement, we observed that 3 PRE biopsies (Pt04, Pt07, and Pt08) displayed an architecture of multiple layers of LRRC15^+^ CAFs lining tumor cell islets, which disappeared after treatment (Figure 5F). These 3 tumors showed an increased CD8^+^ T cell infiltration, as seen through both tumor/stroma ratio and tumor nest density, revealing a more homogeneous distribution of CD8^+^ T cells after treatment (Figure 5F). These CD8^+^ T cell results were confirmed in the NIH validation cohort (Supplemental Figure 6, E and F), where the 3 patients (NIH Pt02, Pt10, and Pt12) with an LRRC15^+^ CAF capsule and CD8^+^ T cell exclusion before BA treatment showed a higher CD8^+^ T cell intratumor penetration (tumor/stroma ratio and tumor nest density) after treatment.

We used scRNA-seq data to further investigate the changes in the CD4^+^ and CD8^+^ T cell subsets, which were annotated using ProjecTILs reference maps (Supplemental Tables 6 and 7) (30). We identified exhausted (CD8.TEX) and progenitor-exhausted (CD8.TPEX) CD8^+^ T cells as the major producers of IFN-γ (Supplemental Figure 7, A and B, and Supplemental Table 7) (30). While CD8.TPEX cells made up a negligible fraction of total CD8^+^ T cells, CD8.TEX cells were the most enriched subset among CD8^+^ T cells in all samples (Supplemental Figure 7D). Notably, the average expression of IFN-γ by CD8.TEX increased in all patients after treatment, emphasizing the reinvigorating capacity of BA (Supplemental Figure 7E).

Together with our prior studies (4, 6), these results suggest that PD-L1/TGF-β blockade therapy, even over a short term, can alter the CD8^+^ T cell–restricting CAF barrier.

### Maintenance of LRRC15^+^ CAFs around a subset of BA-resistant tumor cells in the major responder patient.

To extend our knowledge on the biological impact of BA on the TME upon response, the whole surgical specimen of the MPR (Pt07, PathR 54%) was analyzed through both multiplexed imaging and spatial transcriptomics (Figure 6). As previously mentioned, the Pt07 PRE biopsy had the most detected oncogene alterations and the highest TMB, a high PD-L1 immune positive score, and a higher number of immune aggregates (Figure 2, B–D). The tumor harbored LRRC15^+^ CAF layers surrounding tumor cells, along with a CD8^+^ T cell exclusion, that were both counteracted upon treatment (Figure 5, C–F).

Most of the MPR tumor resection posttreatment consisted of regions of major tumor cell death (Figure 6A) with a global infiltration of T cells into the stroma and tumor cell islets (Figure 6B). A minor area was marked by intact cancer cells (hereafter, BA-resistant cells) exempt from immune infiltration (Figure 6, A and B). In this region, we observed capsule maintenance of densely layered LRRC15^+^ CAFs (Figure 6B). Given that BA treatment induced a massive reduction in LRRC15^+^ CAFs in all patients (Figure 5, C and D), we sought to investigate the specificities of this resistant tumor region. The resistant cells stained positively for pan-keratin (albeit weakly), podoplanin (PDPN), and MCAM, but not for vimentin (Supplemental Figure 8A). A pathologist confirmed that these tumor cells were morphologically poorly differentiated (Figure 6A, zoom). Note that such poorly differentiated cancer cells (e.g., PDPN^+^, MCAM^+^, and KER^lo^) were already observed in the PRE biopsy of this patient, indicating their presence before treatment (Supplemental Figure 8B).

To dissect the potential molecular mechanisms underlying LRRC15^+^ CAF maintenance, we further characterized the differences between the BA-resistant cancer cells and those in areas of major cell death. We performed MERFISH (multiplexed error-robust fluorescence in situ hybridization) spatial transcriptomics with a customized 300-gene panel (Supplemental Table 8) and Xenium spatial transcriptomics with a predesigned 289-gene panel (Supplemental Table 9) on the MPR surgical specimen. Both techniques provided similar results. Unsupervised clustering identified 2 distinct clusters of cancer cells, corresponding to the tumor cells in the area of CAF maintenance and in the region of cell death (Figure 6C). Based on DEG analyses, the BA-resistant cancer cells were characterized by higher expression of stemness-related transcription factors (e.g., *SNAI2*, *SOX9*, *LGR5*, *LGR6*, and *ETV5*) and mesenchyme-associated genes (e.g., *MMP2*, *CLDN11*, and *NID1*), as well as lower levels of genes associated with keratinocyte differentiation (e.g., *CDH1* and *KLF4*) (Figure 6D, Supplemental Figure 8C, Supplemental Tables 10 and 11). Despite the greater presence of LRRC15^+^ CAFs around them, they expressed lower levels of *TGFB1* than their BA-sensitive counterparts (Supplemental Figure 8C). Finally, BA-resistant cancer cells expressed both *FGF3* and *FGFR1*, suggesting an autocrine signaling loop (Supplemental Figure 8C). Although at a lower level than on the BA-resistant cancer cells, *FGFR1* was also detected on the surrounding LRRC15^+^ CAFs (Supplemental Figure 8C), indicating potential crosstalk between the 2 cell types.

The spatial transcriptomic analysis allowed us to identify tumor cells in resistant and sensitive areas. We next analyzed the scRNA-seq data from Pt07 cancer cells (Figure 6E), which provided no spatial information yet deeper information on the transcriptomic profile. We identified 2 clusters of cells that we assigned to resistant- and sensitive-like cancer cells, as they were enriched in the expression of the signatures identified by spatial transcriptomics (Figure 6E, left). scRNA-seq DEG analyses identified similar highly expressed genes compared with Merfish/Xenium data, with higher expression of differentiation genes (e.g., *KRT19* and *FOXA1*) in sensitive-like cells and increased levels of stemness/epithelial–mesenchymal transition–related genes (e.g., *HOXA9*, *WNT6*, *PODXL*, and *LGR5*) in the resistant ones (Figure 6E, right, and Supplemental Table 12). We further pursued scRNA-seq analysis to investigate crosstalk from cancer cells to fibroblasts potentially driving LRRC15^+^ CAF maintenance. Using CellChat (31) and filtering for interactions specific for resistant-like tumor cells and CAFs, we identified 3 predicted communication networks, including FGF signaling (*FGF3*/*FGF19* [cancer cells]–*FGFR1* [CAFs]), in line with our results from spatial transcriptomics (Supplemental Figure 8C); PDGF signaling (*PDGFA* [cancer cells]–*PDGFRA/B* [CAFs]); and GAS signaling (*GAS6* [cancer cells]–*AXL* [CAFs]) (Figure 6F and Supplemental Table 13). These ligand-receptor pairs were specifically highly expressed by resistant-like cancer cells and CAFs, but not sensitive-like cancer cells. Together, these data showed that TGF-β blockade does not systematically affect LRRC15^+^ CAFs; here, their specific localization around highly distinct, poorly differentiated tumor cells suggests that tumor-derived signals may drive the preservation of the LRRC15^+^ CAF capsule despite BA treatment.

## Discussion

Here, we took advantage of the neoadjuvant setting, allowing for the analysis of treatment-induced changes within a short period of time. We provide a deep spatial and molecular dissection of changes induced by combined TGF-β and PD-L1 blockade in patients with locally advanced, untreated HNSCC.

By comparing our results with similar scRNA-seq data from clinical trials targeting the PD-1/PD-L1 axis alone in HNSCC patients, we discovered that TGF-β inhibition had a profound impact on mesenchymal cells. It especially altered LRRC15^+^ CAFs, identified here with a signature (*MMP11, COL10A1*, *COL11A1*, *COL12A1*, *CTHRC1*, and *LRRC15*) highly overlapping with LRRC15^+^ myCAFs described in other human cancers and correlated with poor response to PD-L1 blockade (14, 15).

Inhibition of TGF-β has been explored as a strategy to modulate CAF activity and potentially repolarize CAFs into a less activated state (32). Here, we observed a massive LRRC15^+^ myCAF reduction, yet no significant increase in stromal coverage of IL24^+^CD10^+^ iCAFs, and a strong stromal compartment decrease in a fraction of tumors, confirmed in an independent cohort and through spatial transcriptomics. Our data suggest that TGF-β inhibition combined with PD-L1 blockade in human patients can induce fibroblast depletion rather than a fibroblast polarization shift only. In addition to strongly reducing LRRC15^+^ CAF abundance, 3 weeks of BA treatment was sufficient to substantially remodel the matrix architecture, including partial loss of collagen I and XII fibers. These results are in line with recent work where FAP^+^ CAF targeting using CAR T cells induced a disruption of the desmoplastic stroma within days in mouse pancreatic tumors (8).

T cell marginalization in the peritumoral stroma can limit antitumor response (33, 34). We observed an increase in CD8^+^ T cell infiltration into tumor cell islets in tumors initially immune excluded and harboring an architecture of peritumoral LRRC15^+^ CAF layers. In addition to TGF-β blockade–related CAF capsule rupture, other effects of the treatment could contribute to the increased T cell penetration. For instance, increased *CXCL9* and *CXCL10* expression, likely due to IFN-γ upregulation in CD8^+^ T and NK cells, was observed in DCs and macrophages as well as in stromal and cancer cells. Both the increased IFN-γ signaling and the reduced TGF-β signaling that we observed are in accordance with mouse model preclinical studies on the effects of BA (20). While we cannot rule out the contribution of TGF-β blockade to these results, such changes have been observed when induced by immune checkpoint inhibition alone (35).

Additional BA effects observed on immune cells included a reduction in stromal CD4^+^ Tregs, in agreement with the results from Redman et al. (23) and with the widely reported importance of TGF-β for the functionality and maintenance of Tregs (36). We also found a significant decrease in CD4^+^ conventional T cells, which should be considered in further studies blocking TGF-β, given the antitumor potential of CD4^+^ T cells (37).

The NCT04428047 study could not conclude on the clinical endpoints due to early trial interruption. The similar window-of-opportunity NIH trial of Redman et al. (23) (preoperative D1-D14 BA injections in patients with HPV^–^ HNSCC) found no correlation between PathR and RECIST (Response Evaluation Criteria in Solid Tumors) outcomes and had 71% (10/14) progressive disease (PD) and 29% (4/14) stable disease (SD). Similarly, we found no correlation and report 4/6 PD and 2/6 SD. Notably, the MPR with 54% PathR was considered PD based on RECIST criteria, which could be due to the massive immune infiltration often observed at early phases of immunotherapies (38). A similar pattern of radiological pseudoprogression at the first restaging time point, followed by a durable PR based on RECIST criteria, has been reported in a patient with advanced non–small cell lung cancer treated with BA and was due to documented immune cell infiltration and tumor necrosis (39). Thus, RECIST assessments after 2 doses may not be a reliable indicator of treatment efficacy. The short observation period we used (3 weeks) likely contributed to the lack of RECIST response, as a median time to response in first-line immunotherapy for HNSCC ranges from 1.90 to 3.09 months (40). The limited number of cases in our phase II trial and in Redman et al. (23) restricts definitive conclusions about the BA treatment response rate.

An effect of BA treatment on the TME was observed in all patients. The heterogeneity in PathRs indicates that stromal remodeling alone is unlikely to be sufficient to drive tumor regression. Additional factors, including tumor‑intrinsic features, antigenicity, and the functional state of infiltrating immune cells, likely influence clinical outcomes. In line with this, the major responder patient was distinguished from the others by having a slightly higher TMB, more genomic aberrations, higher PD-L1 on immune cells, and the presence of multiple lymphoid aggregates in the PRE biopsy, suggesting an overall higher immunogenicity at baseline and an increased potential to respond to immunotherapy. The addition of TGF-β blockade might have synergized with checkpoint inhibition by disrupting the LRRC15^+^ CAF capsules surrounding tumor nests in this patient’s PRE biopsy. Additional ancillary studies are required to draw robust conclusions regarding correlations between stromal remodeling and patient outcomes as well as to assess the long-term consequences of such tumor stroma disruption.

The biologic and/or clinical responses observed within such a short time frame (here, only 3 weeks) and for other tumor types (39, 41, 42) underscore the need to find biomarkers that can identify patients who will benefit or not from BA. Our findings suggest that patients with CD8^+^ T cell–excluded tumors and a fibrotic stroma may particularly gain from aPD-L1/TGF-β combination strategies.

## Methods

### Sex as a biological variable.

The NCT04428047 trial included both male (*n* = 4) and female (*n* = 3) patients. However, given the small sample size, the study was not powered to assess sex‑specific differences, and no formal analysis by sex was performed.

### Patients and study protocol.

The patients included in this study were enrolled in a clinical trial entitled “A phase II trial assessing BA, a bifunctional fusion protein targeting TGF-β and PD-L1, in a pre-operative setting for resectable and untreated head and neck squamous cell carcinoma (ICING),” registered under EudraCT number 2019-004052-11 and NCT04428047. The key inclusion criteria were as follows: (a) presenting with a resectable untreated HNSCC of the oral cavity, oropharynx, larynx, and/or hypopharynx, (b) aged 18 years or older, and (c) staged T2N1–3 and T3–4 at any N at M0 (as per the AJCC manual, 7th edition). The full study protocol is available in the supplemental material. Notably, this trial was abruptly ended by EMD Serono due to low outcome improvement in a distinct phase III trial using BA (see supplemental material).

The study described herein was a window-of-opportunity, prospective, single-arm trial of BA approved by the ethical committees of the Institut Curie (6 patients: Pt02, Pt03, Pt04, Pt05, Pt07, and Pt08) and the Institut de Cancérologie de Lorraine (1 patient: Pt11) and sponsored by Unicancer. Seven patients with HNSCC were included (5 oral cavity, 2 oropharyngeal), with a median age of 61 years (range 34–74 years); of these, 6 received the full treatment scheme. All were HPV^–^ as assessed by RT-PCR. One oropharyngeal tumor (Pt07) stained positive for p16, a protein often used as a surrogate marker for HPV^+^ carcinoma, yet thereafter was confirmed to be HPV^–^ by touchdown general primer (GP6^+^) PCR.

There were 3 nonresponders; of these, 2 (Pt08 and Pt11) presented with clinically obvious PD, with Pt08 already noted as having a fast-growing tumor prior to BA treatment. The 2 patients with PD died at month 6 of multiple metastases and local recurrence, respectively. Pt11 (from Institut de Cancérologie de Lorraine) had no sample available for this study and received only 1 injection of BA. Therefore, this case was not further studied for the ancillary analysis. Pt05, with PR and with a cT3N3 tumor, developed lung metastases at month 6 and brain metastases at month 17; this patient died at month 25. At the time of writing, 4 patients were alive without recurrence (nonresponder Pt03 at month 30, PR Pt02 at month 32 and Pt04 at month 22, and MPR Pt07 at month 31). As commonly seen in short-term, immunotherapy-only neoadjuvant trials (3 weeks) (24), no tumor regression was observed, as per RECIST v1.1 criteria.

BA was overall well tolerated, and no surgery was delayed or canceled due to adverse events (AEs) (Supplemental Table 14). The pre- and postoperative courses were as expected in this population. Most AEs were grades 1 and 2, with anemia, asthenia, and inflammatory gingivitis the most frequently observed. Bleeding, a known potential AE of BA (43), occurred in 1 patient (Pt08) and was clinically manageable. Seven grade 3 severe AEs were reported in 5 patients, including 2 cases of anemia and 1 case of disease progression that led to treatment interruption after the first dose (Pt11). Pt08’s death at month 6 was reported as a grade 5 AE.

Race and ethnicity data were not collected. The study was conducted in France, where collection of such data is not routinely performed in clinical trials and is subject to specific legal and ethical constraints. Demographic characteristics collected for study participants included age and sex, which are reported for all enrolled participants.

### Sample processing.

PRE biopsies were taken during the baseline endoscopy, and POST biopsies were taken in a similar manner using biopsy forceps on the day of the surgery during an initial endoscopy performed before starting the resection. Therefore, there were no differences related to sampling method or ischemia time between PRE and POST samples.

The samples came from patients treated at the Institut Curie hospital center with the following IDs: 002, 003, 004, 005, 007, and 008 (hereafter, Pt02, Pt03, etc.). POST Pt05 sample failed for experimental reasons. All fresh samples were processed immediately after sampling, between March and July 2021.

Fresh tumor samples were mechanically (scalpel) and enzymatically digested in 2 mL of CO_2_ independent medium (18045-054, Gibco) containing 5% FBS (CVFSVF00-01, Eurobio), 200 μL collagenase I (C0130-16, Sigma-Aldrich; 20 mg/mL), 5 μL DNAse I (11284932001, Roche; 10 mg/mL), and 80 μL hyaluronidase (H3506, Sigma-Aldrich; 50 mg/mL) for 50 minutes at 37°C in a shaker, with 2 rounds (at 15 and 30 minutes of incubation) of rapid up and down pipetting using a 10 mL serological pipette. Samples were then washed in PBS (PBS Dulbecco; CS1PBS01-01, Eurobio) enriched with 0.4% EDTA (0.5 M EDTA ultrapure, pH 8.0; 155750020, Thermo Fisher Scientific) and 1% human serum (H4522, Sigma-Aldrich) and then filtered (40 μm filters; 22363547, Fisher Scientific) and resuspended in PBS enriched with 2% FBS and 1 mM CaCl_2_.

The stem cell kits were used to remove first red blood cells and then dead cells, as recommended by the manufacturer (18170, EasySep RBC Depletion Reagent Kit; 17899, EasySep Dead Cell Removal [Annexin V]; and 18102, EasyPlate EasySep Magnet; all STEMCELL Technologies). Cells were resuspended at 700 cells/μL in DMEM (61965-026, Gibco) in 10% FBS. Single-cell sequencing was performed using the Single Cell 3′ V3 kit as per the manufacturer’s protocol (10x Genomics), with 16,000 cells loaded for an expected recovery of 10,000 cells per sample. Sample quality was confirmed using a high-sensitivity DNA chip and the 2100 Bioanalyzer (Agilent Genomics). Samples were sequenced at a depth of 50,000 reads/cells on a NovaSeq platform (Illumina).

### scRNA-seq data processing and analysis.

The POST sample from Pt05 was excluded directly after sequencing due to insufficient quality. All other samples underwent quality control (QC) on the FASTQ files using FASTQC and MultiQC, controlling for (a) sequencing quality per base and sequence, (b) per base sequence and GC content, (c) sequence lengths, (d) N content, (e) adapter content, and (f) overrepresented sequence. For read alignment and unique molecular identifier (UMI) quantification, CellRanger software v5.0.0 was used on Human Cell Ranger reference 2020-A (human GRCh38; GENCODE v32/Ensembl98), and a post-mapping QC was performed using the Cell Ranger pipeline (10x Genomics).

True cells were distinguished from empty droplets in the Cell Ranger pipeline. An initial total of 170,482 cells was obtained. Cells were filtered using the combination of the following methods and thresholds: 14,863 cells were identified as having < 800 UMIs/cell (threshold based on data distribution); 39,882 cells were identified as expressing < 600 genes per cell (threshold based on data distribution); and 20,075 cells were identified as expressing > 20% of mitochondrial genes. DoubletFinder (R package) was used to filter out an additional 9,739 potential doublet cells.

The filtered count matrices were analyzed by Seurat R version 4.0.5 (44). The SCTransform normalization method was applied and used for visualization and clustering analysis. Graph-based clustering was performed with the Seurat R package and represented by UMAP. The optimal number of principal components (PCs) used to identify clusters was determined based on curve inflection on the elbow plot representing the decay in explained variance per additional PC. The optimal resolution was determined using a combination of cluster stability observed on Clustree 0.5.0 (45); expected cell types and states were based on previous knowledge, homogeneity of top gene expression within each cluster, and distribution across samples. The selected number of PCs and resolutions are detailed in Supplemental Table 15. Marker genes defining each cluster were identified using the SeuratAllMarkers function with Wilcoxon’s rank sum test using a minimal log_2_FC of 0.25. Ten clusters were identified as corresponding to the main cell populations expected in cancer, except neutrophils. Two cancer cell clusters emerged, 1 containing cells from all patients and 1 containing cells from Pt07 only (which was excluded from the global change analysis for patients due to its patient specificity).

Each cluster was further subclustered using the same methodology for more detailed analyses. After subclustering, some subclusters were defined by strong signatures of contamination by macrophages, cancer cells, or T cells; these were excluded from subsequent analysis. Major cluster 5 was reclustered after having excluded contaminated subclusters and genes contributing to technical noise (XIST, mitochondrial genes, and ribosomal genes).

The final number of cells of the ICING count matrix is detailed in Supplemental Table 3.

### Fibroblast subclustering.

Initial subclustering of major cluster 5 yielded 10 clusters, of which 4 were obvious doublets expressing a signature of macrophages, T cells, or cancer cells despite earlier doublet cell removal. To avoid these confounding effects on fibroblast subset identification, these were excluded. For the analysis, the remaining cells were subclustered, from which we identified 2 clusters of CAFs and 1 cluster of pericytes.

### T cell subclustering.

ProjecTILs (30) was used to annotate CD4^+^ and CD8^+^ T cell subsets, using the label transfer function of pan-cancer CD4^+^ and CD8^+^ TIL atlas reference maps and default parameters. The reference maps can be found at https://doi.org/10.6084/m9.figshare.23608308 (human CD8^+^ TILs) and https://doi.org/10.6084/m9.figshare.21981536.v1 (human CD4^+^ TILs).

### Pseudobulk analysis.

To investigate global changes induced by BA that were independent of patient or response, a pseudobulk analysis was performed with strict thresholds to increase the probability of isolating real effects from noise. Plasma cells and mast cells were excluded due to contamination with macrophage genes, and cancer cells 2 were excluded as they were only present in Pt07. Pt05 was excluded due to the lack of a POST sample. The pseudobulk count matrix was established with the AggregateExpression function in the Seurat package (44), aggregating RNA counts by patient, cell type, and PRE/POST status.

DESeq2 (46) was used to compute the DEGs pre- versus posttreatment. The generalized linear model was set to control for the effect of patient and cell type, making it a paired analysis per cell type per patient. *P* values were adjusted using the Benjamini-Hochberg method (47). Log_2_FC values of each gene were adjusted for the information content using the lfcShrink function (48).

The following parameters were used to filter for relevant genes: genes expressed in at least 15% of cells per cell type in 3 out of 5 PRE or POST samples were kept. The logFC cutoff was ±0.25, and the adjusted *P* value cutoff was ≤0.05. Ribosomal, mitochondrial, and immunoglobulin genes, as well as the gender-specific gene *XIST*, were excluded (Supplemental Table 16).

The top 50 up- and downregulated genes were then plotted onto the major cell types in a heatmap using ComplexHeatmap (49) by plotting the mean expression value per cluster per PRE/POST sample using the AverageExpression function in Seurat.

### Functional enrichment analysis.

Overrepresentation analysis (ORA) of DEGs in collections of gene sets was performed using the clusterProfiler package (version 4.10.0) (50). The org.Hs.eg.db gene set database package version 3.18.0 (51) was used with the enrichGO(), enrichKEGG(), and enrichWP() functions to perform ORA with Gene Ontology, KEGG, and WikiPathways gene sets, respectively. The msigdb database package version 7.5.1 (52) was used to retrieve REACTOME (53) and cancer module gene sets for ORA with the enricher function. The default Fisher’s exact test was used, and the generated *P* values were corrected using the Benjamini-Hochberg method.

### ORION multiplex IF.

Dewaxing and antigen retrieval prior to staining were performed with EnVision FLEX Target Retrieval Solution, High pH (Dako) for 15 minutes at 95°C. Retrieval solution excess was removed via 2 washes in a PBS bath. Slides were then immersed in quench buffer (4.5% H_2_O_2_, 24 mM NaOH, and 1× PBS), exposed to white light (1 hour), and transilluminated with UV light (30 minutes). To avoid fluorescent background, sections were incubated for 15 minutes with Image-iT FX Signal Enhancer (Thermo Fischer Scientific) and rinsed with PBS-T (0.025% Triton X-100 and PBS) after this and each following step.

Slides were first stained with unlabeled primary antibodies against TCF1, FOXP3, and αSMA for 1 hour at room temperature and then incubated with ArgoFluor-conjugated secondary antibody for 30 minutes at room temperature. Slides were stained with all other ArgoFluor-conjugated primary antibodies for 2 hours at room temperature, incubated with Hoechst 33342 (H3570, Thermo Fisher Scientific) for 30 minutes, rinsed, mounted on coverslips using ArgoFluor mounting medium (Rarecyte), and stored at 4°C until acquisition. Acquisition was performed with the Rarecyte Orion instrument. All antibodies and further details are given in Supplemental Table 17.

### Multiplex IHC.

The multiplex IHC consecutive staining on a single slide technique was performed as previously described (54), with some adjustments. Tissues were baked at 60°C for 1 hour, deparaffinized in xylene (10467270, Fisher Scientific), and rehydrated. Heat-induced epitope retrieval was done in citrate buffer (pH 6.1; S169984-2, Dako) or EDTA buffer (pH 9; S236784-2, Dako) in a 95°C water bath for 30 minutes for the first staining, and for 15 minutes for the following staining, followed by incubation in REAL peroxidase blocking solution (S202386-2, Agilent Dako) for 10 minutes. If the primary antibody was the same species as any antibody used in prior stains, another blocking step was added with Fab Fragment for anti-rabbit (711-007-003, Jackson ImmunoResearch Europe Ltd.) and anti-mouse (715-007-003, Jackson ImmunoResearch Europe Ltd.) antibodies for 20 minutes. Serum-free protein block (X090930-2, Agilent Dako) was added for 10 minutes. Primary antibodies were incubated between 1 and 2 hours at room temperature and then detected with a secondary antibody conjugated with horseradish peroxidase (anti-rabbit: K400311-2, Agilent Dako; anti-mouse: K400111-2, Agilent Dako; anti-goat: VC004-050, R&D Systems; anti-rat: VC005-050, BioTechne) followed by chromogenic revelation with 3-amino-9-ethylcarabazole (K3468, Agilent Dako). Slides were counterstained with hematoxylin (6765001, Thermo Fisher Scientific) and mounted with Glycergel aqueous mounting medium (C0563302, Dako). After scanning (C13220-04, NanoZoomer S360 digital slide scanner, Hamamatsu), tissues were bleached with ethanol baths, and the next staining cycle was performed starting with the heat-induced epitope retrieval. Hematoxylin staining was utilized to align the overlays in HALO software (Indicalabs) and to merge them into single multichannel image files (ome-tif). All antibodies and further details are given in Supplemental Table 18.

### TSA Opal 6 plex IF.

Matched, paired samples from 6 patients (NIH cohort) (23) with HPV^–^ HNSCC and treated with BA (baseline and on treatment) were fixed, paraffin embedded, and sectioned. Tissue sections were immunostained using a validated multiplex IF panel with the TSA Opal 6 plex kit (Quanterix) following the manufacturer’s protocol. The panel included LRRC15, collagen I, CD3, CD8, Ki67, CK, and DAPI. Deparaffinization, rehydration, epitope retrieval, and staining were performed on the BOND RX Autostainer (Leica Biosystems). Optimal staining conditions for each antibody were established using standard IHC and single IF prior to panel assembly. Details on the antibodies, staining protocol, and the Opal kit used are provided in Supplemental Table S17. Whole-slide imaging was performed using the PhenoImager HT scanner (Quanterix) at ×20 magnification.

### Image analysis and cell type calling.

Tumor and adjacent/healthy tissue areas were annotated by a pathologist. Thereafter, only tumor areas were analyzed, as not all samples contained nontumor tissue areas. Multichannel images were analyzed in QuPath (55). Cells were detected using the StarDist for QuPath extension (56). Subsequently, classifiers were trained for the various cell types to be detected using the random tree classifier included in QuPath. All stainings were used to train the classifiers. As some unspecific CD4 staining was observed in multiple samples, only CD3^+^CD45^+^ cells were considered when classifying T cells to avoid false positives. CD8^+hi^ CD4^+lo^ cells were considered CD8^+^ T cells. The signal-to-noise ratio of the PD-1 staining was too low to be reliably analyzed and was thus excluded from the analysis. Pixel classifiers were trained to distinguish tumor islets and stromal area.

IF images of the NIH tumor samples generated using TSA Opal were used to train a classifier with the DenseNet V2 plug-in (Halo, Indicalabs) to discriminate tumoral and stromal compartments. An LRRC15 classifier was trained within the stromal compartment to determine the coverage of LRRC15^+^ fibroblasts. Nuclear segmentation was performed using an AI custom nuclear segmentation type with a maximum cytoplasm radius of 1. CD8^+^ and CD3^+^ T cell populations were identified based on a positive intensity threshold. The areas of each compartment (stroma and tumor) were used to calculate CD8^+^ and CD3^+^ T cell densities (cells/mm^2^).

### HPV typing.

HPV typing was first performed at the pathology department via Sybr Green–based real-time PCR with primers specific for HPV16, HPV18 and HPV33. Second, HPV L1 amplicons for patients negative for the aforementioned HPV types were generated using GP6^+^ touchdown PCR and sequenced by Sanger sequencing, and the results were aligned to HPV reference sequences using NCBI nucleotide blast.

Due to the p16^+^ status of Pt07, the Molecular Genetics platform at Institut Curie carried out another round of HPV detection via hybridization-based target enrichment, as previously described (57), which yielded a negative result.

### Targeted NGS on tissues.

Genetic alterations were detected via targeted NGS on a 571-gene panel (Supplemental Table 19), and the TMB was calculated by the Cancer Molecular Genetics platform, as described previously (25). Briefly, DNA was extracted from FFPE tumor biopsies, and 50 ng of DNA was used to establish a library using the Agilent Sureselect XT2 library prep kit according to the manufacturer’s instructions. The pooled libraries were sequenced on a NovaSeq 6000 (Illumina) S2′150 bp flow cell. Reads were then mapped to the human reference genome (hg19) using the Burrows-Wheeler Aligner (58) with default settings. After further quality control, variants were called using a combination of SAMtools (59) and Varscan2 (60) and then annotated using ANNOVAR-provided databases. Variants presenting ± 10 bp from exon–exon junctions were annotated as splice site mutations.

Variant quality was controlled for coverage, and irrelevant variants were removed by a minimum allelic ratio threshold of 5% and a maximal frequency in the population of 0.1%. Based on the Cancer Hotspot Database, truncating and hotspot mutations with a minimal coverage of 200 reads were retained for inactivating mutations of tumor suppressor genes, while known activating missense variants of oncogenes were retained with no minimal coverage.

### MERFISH spatial transcriptomics.

A custom multipurpose 300-gene panel was developed by our group that balanced genes for cell type annotation, immune response, and genes of interest from various sources (e.g., treatment-induced DEGs) (Supplemental Table 8).

Tissue sections (5 μm thick) were adhered to the MERSCOPE beaded slide (Vizgen) and stored in 70% ethanol at –20°C for a maximum of 1 month. FFPE tissues were prepared in a standard manner, following the Vizgen instruction manual, with minor adaptations. In short, a cell boundary staining was applied, followed by hybridization with a custom-made 300-gene panel. The section was then embedded and cleared. For extended clearing of the highly autofluorescent tissue, digestion was prolonged to 6 hours at 37°C, followed by 4 hours of extra concentrated clearing (1:10 of ProtK at 800 units/mL) at 47°C. Directly before imaging, the section was embedded again to avoid tissue lifting during acquisition. Slides were imaged with the MERSCOPE machine and processed with the software version 233. Cell segmentation was performed based on DAPI and membrane staining using the Vizgen post-processing tool and the cellpose algorithm. Data were inspected using the MERSCOPE visualizer software.

Cell segmentation quality was assessed using the mutually exclusive coexpression rate approach and visual inspection. Cells having a total RNA count lower than 5 and a volume lower than 100 μm^3^ were filtered out. Data normalization, dimensionality reduction, and cell clustering were performed using scanpy (version 1.10.3) (61). Cell type density was computed based on 1.5 × 1.5 mm crops. Analyses were performed using SpatialData framework (version 0.1.2) and python (version 3.9.18).

### Xenium spatial transcriptomics.

Tissue sections (5 μm thick) were adhered to a Xenium slide and stored at 4°C for 1 week. FFPE tissues were prepared in a standard manner following the 10′ instruction manual (without adaptations). Briefly, padlock probes from the standard human lung panel (PN 1000601) were hybridized to the slides overnight, followed by rolling circle amplification. Slides were then imaged with the Xenium machine and processed with the analyzer software version 1.6. Cell segmentation was based on DAPI staining only, with an expansion around the staining of 15 μm radius or until the next cell boundary was encountered. Data were inspected using Xenium Explorer and subsequently analyzed using Seurat version 5. Cells having a total RNA count or number of detected genes lower than 10 were filtered out. Data normalization, dimensionality reduction, and cell clustering were performed using Seurat version 5 inside R (version 4.1.2).

### Statistics.

Statistical analyses were performed using R (62) and GraphPad Prism. Data were considered significant for *P* values (or adjusted *P* values, when appropriate) of < 0.05.

### Study approval.

The translational analyses reported in this study were conducted within the ICING clinical trial, a phase II study evaluating BA in patients with resectable, previously untreated HNSCC. The clinical trial was registered under EudraCT number 2019-004052-11 and ClinicalTrials.gov identifier NCT04428047 and was approved by French authorities (Comité de Protection des Personnes and Agence Nationale de Sécurité du Medicament et des Produits de Santé). The study and associated translational research program, including genomic, transcriptomic, and immunomonitoring analyses of blood and tumor samples, were also reviewed and approved by the Comité de Recherche Institutionnel of Institut Curie (approval no. INT200232). All participants provided written informed consent prior to study enrollment, including consent for collection and research use of biological samples and associated clinical data.

### Data availability.

The ICING dataset used in this article contains human genomic, transcriptomic, and associated clinical data generated within the ICING clinical trial (EudraCT 2019-004052-11; ClinicalTrials.gov NCT04428047) and its ancillary study. The scRNA-seq dataset has been deposited in the European Genome-Phenome Archive under accession number EGAC50000001151. Given the potentially identifiable nature of human genomic data, access is controlled. Data requests are managed by Unicancer, the data owner, through a Data Access Agreement. Requests for access should be submitted to mta-dta@unicancer.fr and helene.salmon@curie.fr and will be reviewed by representatives of Unicancer and Institut Curie. The spatial transcriptomic dataset has been deposited in the BioImage Archive under accession number S-BIAD4011. Values for all data points in graphs are available in the Supporting Data Values file.

## Manuscript information

### Author contributions.

CH and JTW contributed equally to this work and share first authorship. Both authors were responsible for data curation, formal analysis, investigation, and drafting/editing of the manuscript. The order of the co–first authors reflects CH’s dual role as both surgeon and researcher in the clinical trial conducted at Institut Curie. MA, JG, BD, AA, VG, IP, WZ, NR, J Merlevede, and AZ: formal analysis. PS: formal analysis, investigation, and writing (review and editing). KJEB, RL, DL, and WL: methodology and investigation. SL: methodology. OC, GM, NB, ADV, GD, LG, JK, and IB: investigation. J Mesple: formal analysis and investigation. MK, LM, and CS: project administration. VG, SJDH, MGD, and AR: writing (review and editing). CTA and JLG: resources. CLT: conceptualization, resources, and writing (review and editing). CH and HS: conceptualization, resources, supervision, funding acquisition, and writing (review and editing).

### AI disclosure.

Microsoft Copilot (GPT-5 chat model) was used in 2026 for language editing, and all AI-generated suggestions were critically reviewed by the authors.

### Conflict of interest.

CH has consulting or advisory roles for Nanobiotix and is currently employed by Owkin Inc. JG has a consulting role for Owkin Inc. MK has a consulting role for AZD and Roche. CLT has participated on advisory boards for MSD, BMS, EMD Serono, AstraZeneca, Roche, GSK, Celgene, Rakuten, Seattle Genetics, Novartis, and Nanobiotix and has consulting role for Owkin Inc.

### Funding.

This study was supported by the following:

- Merck KGaA (CrossRef Funder ID: 10.13039/100009945) supported the ICING clinical trial and associated study and provided BA.
- ARC Emerging Leaders in Oncology supported translational research.
- PSL University IdEx grant (ANR-10-IDEX-0001-02 PSL) supported translational research.
- Centre d’Investigation Clinique IGR-Curie 1428 supported translational research.
- Site de Recherche Intégrée sur le Cancer (INCa-DGOS-Inserm-ITMO cancer_18000) supported translational research.
- The French Government “Investissements d’Avenir” program supported translational research.
- France 2030 program launched by the French Government.
- Fondation pour la Recherche Médicale.
- European Union’s Horizon 2020 Research and Innovation Programme under Marie Skłodowska-Curie grant agreement 847718.
- Regarding the NIH cohort, the National Cancer Institute has a Cooperative Research and Development Agreement with EMD Serono, which provided BA and funding for co-development of this agent.
- Intramural Research Program of the Center for Cancer Research, National Cancer Institute, NIH (ZIA BC 010666).

This work is the result of NIH funding, in whole or in part, and is subject to the NIH Public Access Policy. Through acceptance of this federal funding, the NIH has been given a right to make the work publicly available in PubMed Central.

## Supplementary Material

Supplemental data

ICMJE disclosure forms

Supplemental tables 1-19

Supporting data values

## Figures and Tables

**Figure 1:**
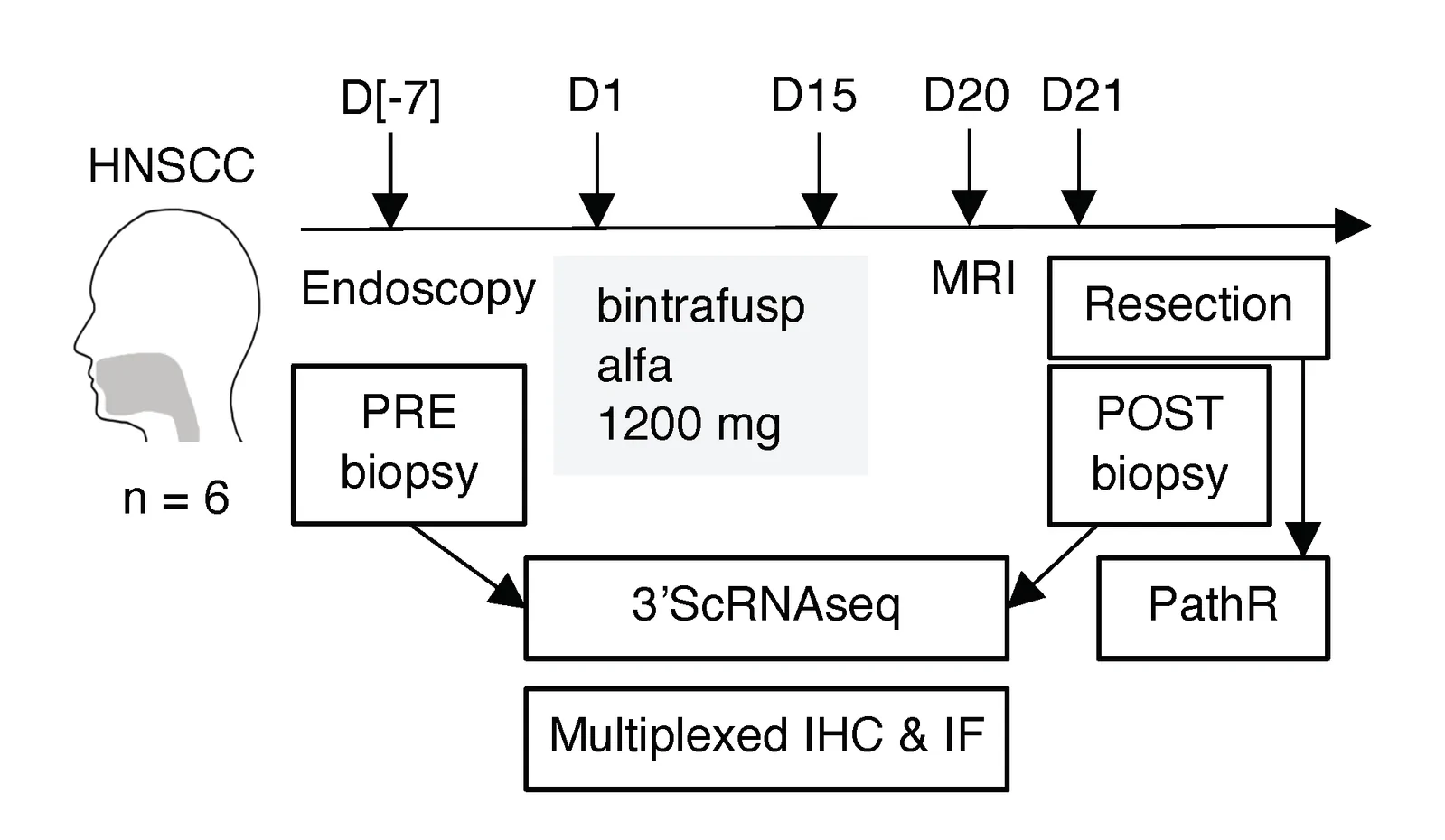
Study overview and patient characteristics. Study scheme and associated analysis on tissue samples.

**Figure 2:**
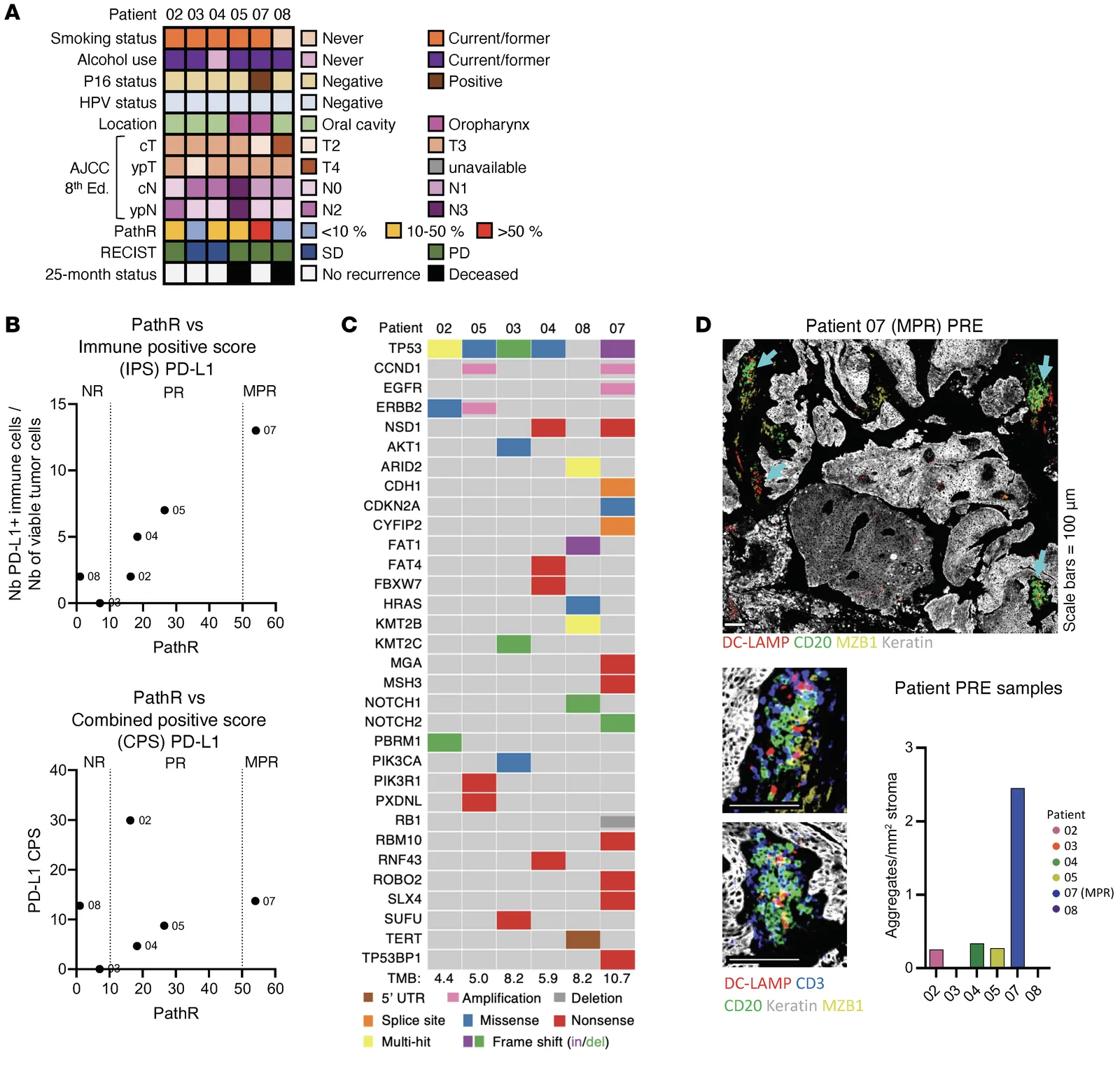
Patient characteristics. (**A**) Characteristics and outcome for each of the 6 patients. c, clinical categorization; yp, post-neoajuvant therapy pathological categorization. (**B**) PathR versus PD-L1 immune positive score (IPS; top) and combined positive score (CPS; bottom) for each patient. NR, nonresponder. (**C**) Genomic alterations and TMB. (**D**) Multiplex IHC staining images highlighting immune cell aggregates in the PRE biopsy of the MPR (Pt07). Bottom right: quantification of aggregates per stromal area in all patients’ PRE biopsies. Scale bars: 100 μm.

**Figure 3:**
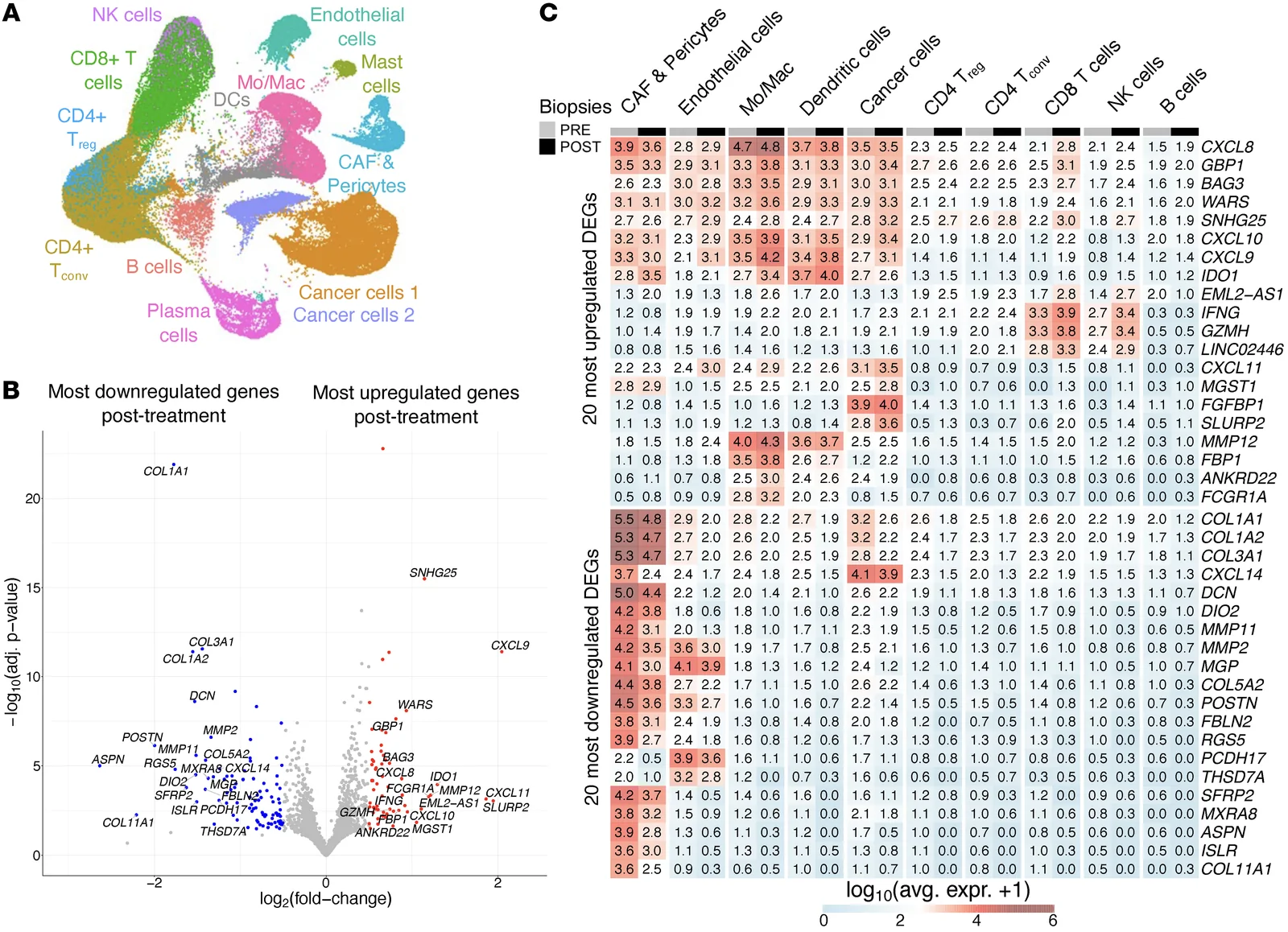
BA treatment reduces TGF-β signaling, with the strongest impact on mesenchymal cells. (**A**) scRNA-seq of 87,645 cells from *n* = 6 PRE and *n* = 5 POST samples after QC filtering projected on a UMAP and clustering of the main cell types. (**B**) Volcano plot showing the results of DEG analysis between PRE and POST samples from 5 paired patients (*n* = 5). Pseudobulk profiles were generated by aggregating cells within each patient and cell type (cluster). Red dots represent upregulated genes, and blue dots represent downregulated genes. The top 20 up- and downregulated genes are depicted (all DEGs are listed in Supplemental Table 4). (**C**) Heatmap showing the gene expression level of the top 20 up- and downregulated DEGs from the analysis in **B**, shown for each cell type in pre- and posttreatment conditions, respectively. Mo/Mac, monocytes and macrophages. Analysis performed with the *n* = 5 paired PRE/POST samples. Values represent log_10_-transformed average expression levels (a difference of 1 corresponds to a 10-fold difference in expression).

**Figure 4:**
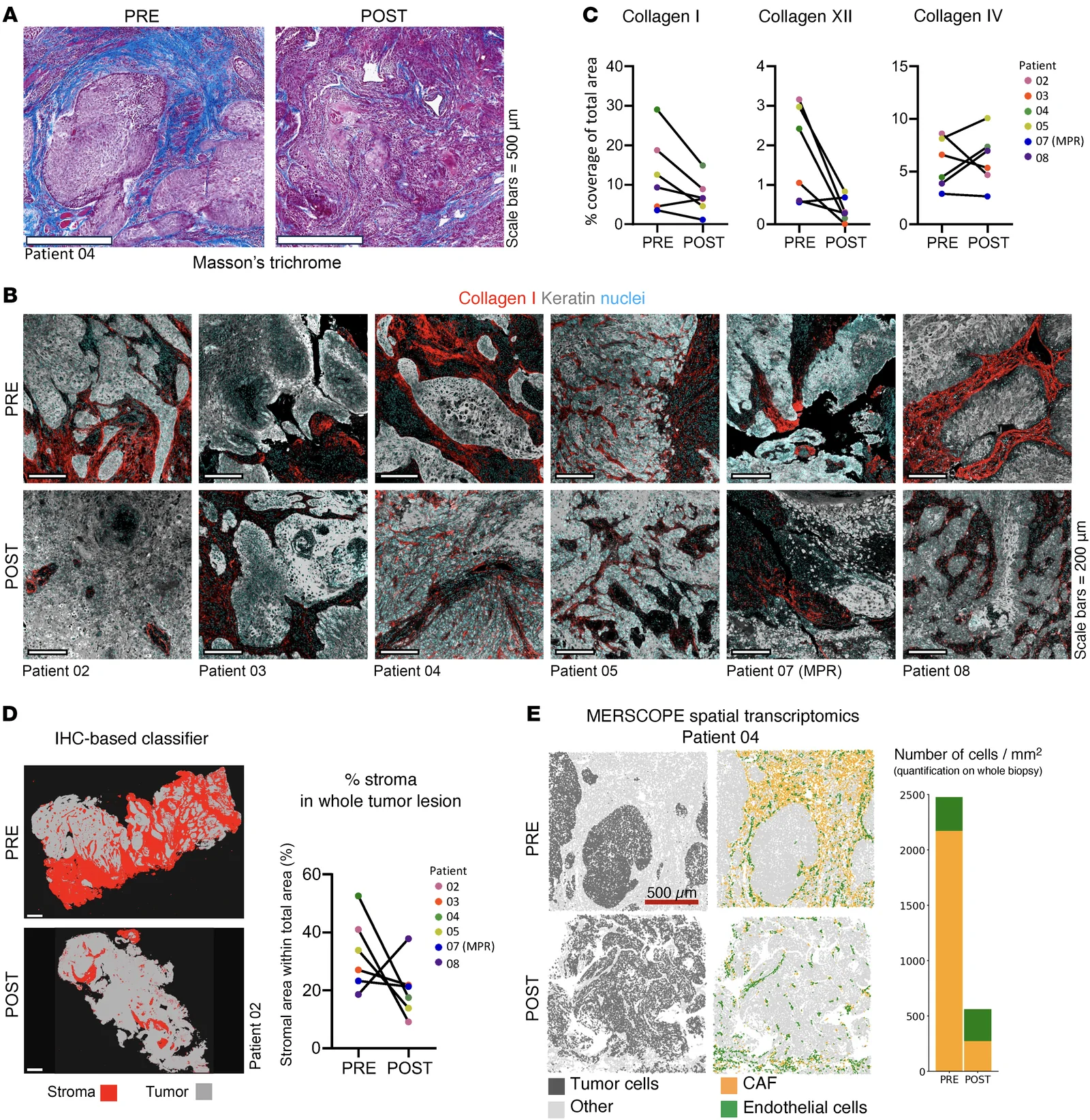
BA induced a strong remodeling of the stromal architecture. (**A**) Representative image of Masson’s trichrome staining highlighting decrease in collagen fiber content (blue) after treatment. Scale bars: 500 μm. (**B**) Collagen I staining of each PRE and POST biopsy. Scale bars: 200 μm. (**C**) Quantification of the surface covered by collagens I, XII, and IV. Wilcoxon’s paired test, *P* values: collagen I = 0.062, collagen XII = 0.062, and collagen IV = 0.844. (**D**) Left: example of tumor and stroma classifier on Pt02 (QuPath analysis). Right: quantification of the area covered by the stromal compartment within the whole tumor lesion for all paired samples. Wilcoxon’s paired test, *P* value = 0.156. Scale bars: 500 μm. (**E**) MERFISH spatial transcriptomics on Pt04 PRE and POST biopsies. Cells were segmented and unbiasedly clustered. Left: visualization of the resulting cancer cell cluster (dark gray), endothelial cell cluster (green), and CAF cluster (yellow). Right: cell density quantification. Scale bar: 500 μm.

**Figure 5:**
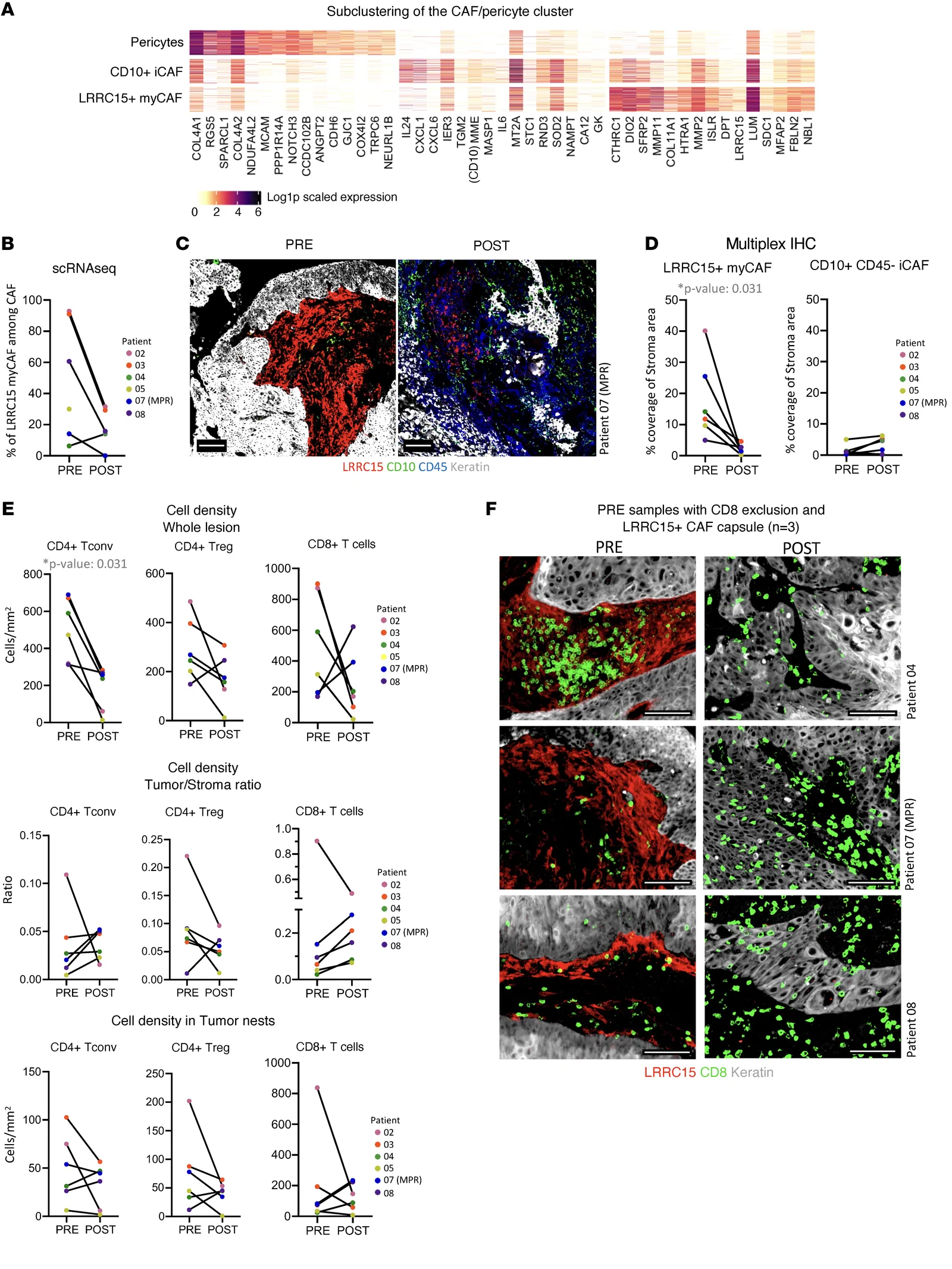
BA induced a systematic decrease in LRRC15^+^-activated CAFs and an increase in intratumor CD8 T cell infiltration in immune-excluded samples with CAF capsule. (**A**) Heatmap of the top 15 DEGs of CAF/pericyte subclusters identified via scRNA-seq. (**B**) Percentage of LRRC15^+^ CAFs among all CAFs within each scRNA-seq sample (pericytes are not counted as CAFs). Wilcoxon’s paired test, *P* value = 0.125 (Pt05 POST scRNA-seq run failed). (**C**) Representative images of PRE and POST biopsies stained for LRRC15, CD10, CD45, and pan-keratin. Scale bars: 100 µm. (**D**) Stromal area coverage of LRRC15^+^ (left) and CD10^+^CD45^–^ (right) CAFs as quantified for each biopsy. Wilcoxon’s paired test, *P* values: LRRC15^+^ myCAF = 0.031; CD10^+^ iCAF = 0.156. (**E**) Quantification of CD8^+^ T cell, CD4^+^ T cell, and Treg infiltration. Wilcoxon’s paired test. *P* values for whole-lesion density: CD4^+^ T cells = 0.031, CD4^+^ Treg = 0.218, and CD8^+^ T cells = 0.313. *P* values for tumor/stroma ratio: CD4^+^ T cells = 0.438, CD4^+^ Treg = 0.219, and CD8^+^ T cells = 0.438). *P* values for density in tumor nests: CD4^+^ T cells = 0.563, CD4^+^ Treg = 0.219, and CD8^+^ T cells = 0.999. (**F**) ORION images from Pt04, Pt07, and Pt08 presenting LRRC15^+^ CAF capsule architecture and CD8^+^ T cell exclusion pretreatment. Right: posttreatment images showing loss of LRRC15 CAFs and increase in CD8^+^ intratumoral infiltration. Scale bars: 100 μm.

**Figure 6:**
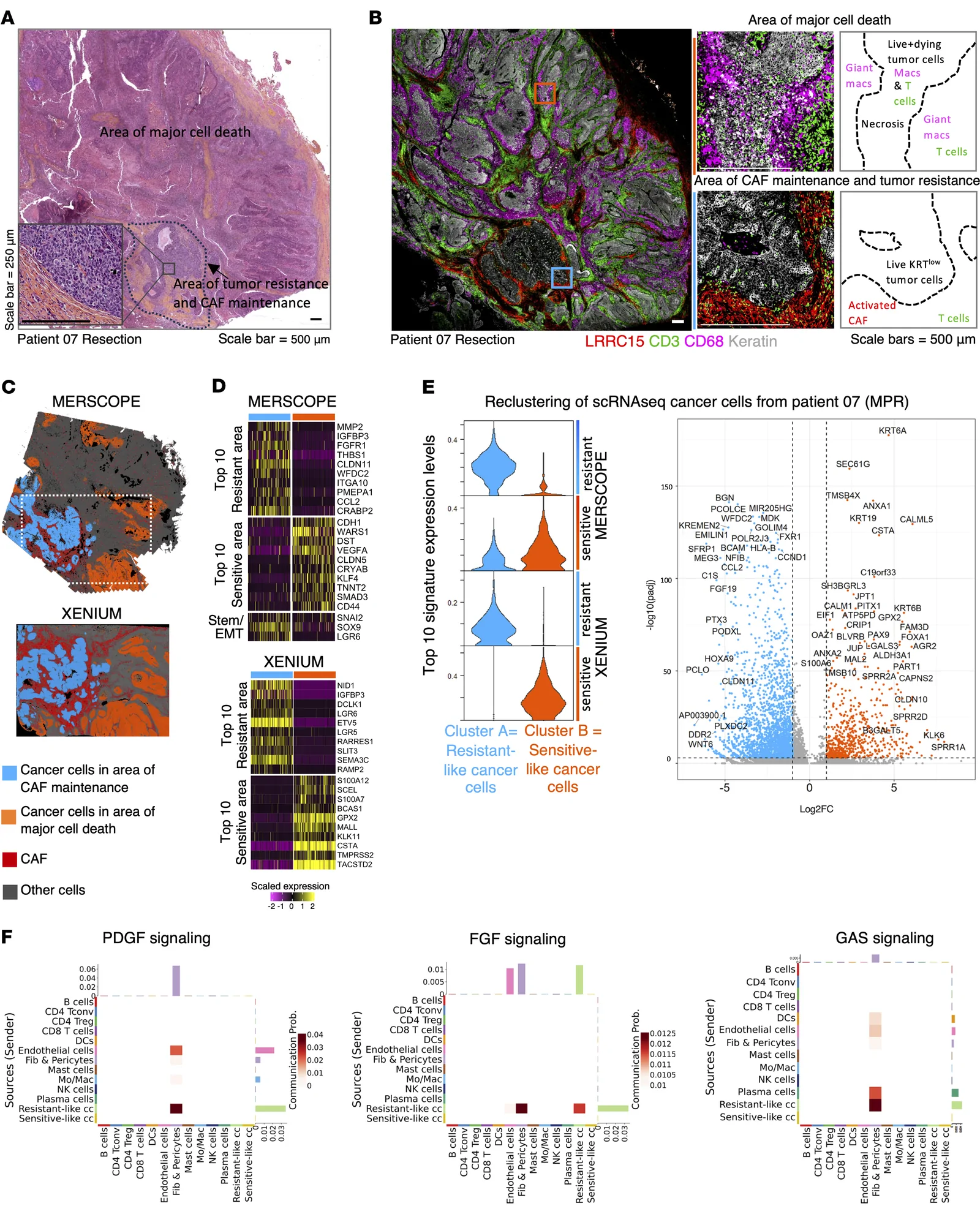
Maintenance of LRRC15^+^ CAFs around a subset of distinct cancer cells in the major responder patient. (**A**) Hematoxylin, eosin, and saffron image of the MPR’s resection (Pt07, PathR 54%), with a representative zoom showing dense fibers surrounding resistant tumor cells. Scale bars: 500 μm, 250 μm (zoom). (**B**) Multiplex IHC image of the MPR’s resection revealing a large area of major cell death with T cell and macrophage infiltration and a minor area of LRRC15^+^ CAF capsule maintenance and immune exclusion. Right: zooms of each area, with illustrative sketches. Scale bars: 500 μm. (**C**) Merscope and Xenium transcriptomic analysis of the MPR posttreatment resection. Cells were segmented and unbiasedly clustered. Visualization of the 2 resulting cancer cell clusters: 1 (blue) in the CAF (red) maintenance area and 1 (orange) in the cell death area. (**D**) Top 10 DEGs between the 2 cancer cell clusters as well as stemness/epithelial–mesenchymal transition–related DEGs. (**E**) Reclustering of all scRNA-seq cancer cells of patient 7 (both pre- and posttreatment cancer cells) unbiasedly gave 2 clusters. Left: enrichment of the top 10 Merscope and Xenium signatures (Figure 6D) in the 2 cancer cell subclusters. Right: volcano plot of DEGs between cluster A (resistant-like) and cluster B cancer cells from the scRNA-seq dataset. (**F**) Ligand-receptor interaction analysis based on the scRNA-seq data (all Pt07 cells included) and the CellChat framework, showing the only 3 signaling pathways identified between resistant-like cancer cells (sender) and fibroblasts (receiver).

## Version 1 11/02/2026

Electronic publication
