# Supplementary material for: Neoadjuvant PD-L1/TGF-β blockade can rapidly disrupt the tumor stroma in head and neck cancer patients
Source: J Clin Invest. 2026 Nov 2;136(21):e198718. doi: 10.1172/JCI198718 (PMC13722384; doi:10.1172/JCI198718)
Supplement: Supplementary file 1 [file jci-136-198718-s079.pdf]

Figure S1

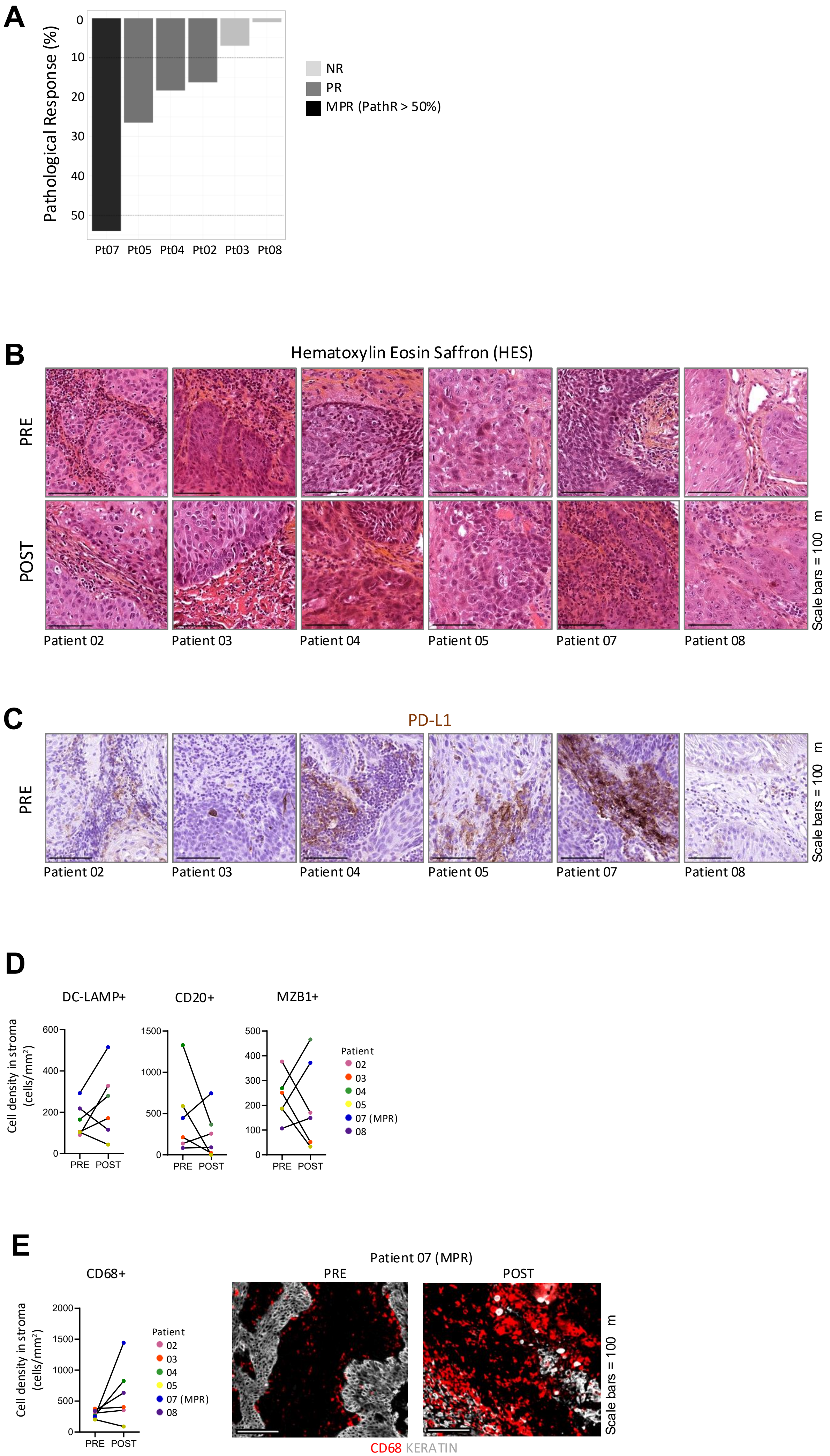

A
Pathological Response (%)
0
10
20
30
40
50
Pt07 Pt05 Pt04 Pt02 Pt03 Pt08
NR
PR
MPR (PathR > 50%)
B
Hematoxylin Eosin Saffron (HES)
PRE
POST
Scale bars = 100 m
Patient 02
Patient 03
Patient 04
Patient 05
Patient 07
Patient 08
C
PD-L1
PRE
Scale bars = 100 m
Patient 02
Patient 03
Patient 04
Patient 05
Patient 07
Patient 08
D
DC-LAMP+
CD20+
MZB1+
Cell density in stroma (cells/mm²)
0
200
400
600
500
1000
1500
100
300
PRE POST
Patient
02
03
04
05
07 (MPR)
08
E
CD68+
Cell density in stroma (cells/mm²)
0
500
1000
1500
2000
PRE POST
Patient
02
03
04
05
07 (MPR)
08
Patient 07 (MPR)
PRE
POST
Scale bars = 100 m
CD68 KERATIN

Supplementary Fig. 1 | A, Waterfall plot showing pathological response (PathR) rates. PR: Partial Response; NR: Non Response; MPR: Major Partial Response. **B,** Representative HES images across the spectrum of pathological responses. **C**, Representative images of the PD-L1 staining of the PRE biopsies of all patients. **D**, Quantification of the stromal infiltration of mature dendritic cells (DC-LAMP+), B cells (CD20+), plasma cells (MZB1+). Wilcoxon paired test, p-values: DC-LAMP = 0.219; CD20 = 0.563; MZB1 = 0.688. **E**, Quantification of the stromal infiltration of macrophages (CD68+) for all PRE and POST biopsies (left; Wilcoxon paired test, p-value: 0.156) and representative multiplex imaging of CD68+ macrophage infiltration in PRE and POST biopsies of Pt07 (MPR). Scale bars: 100μm.

Figure S2

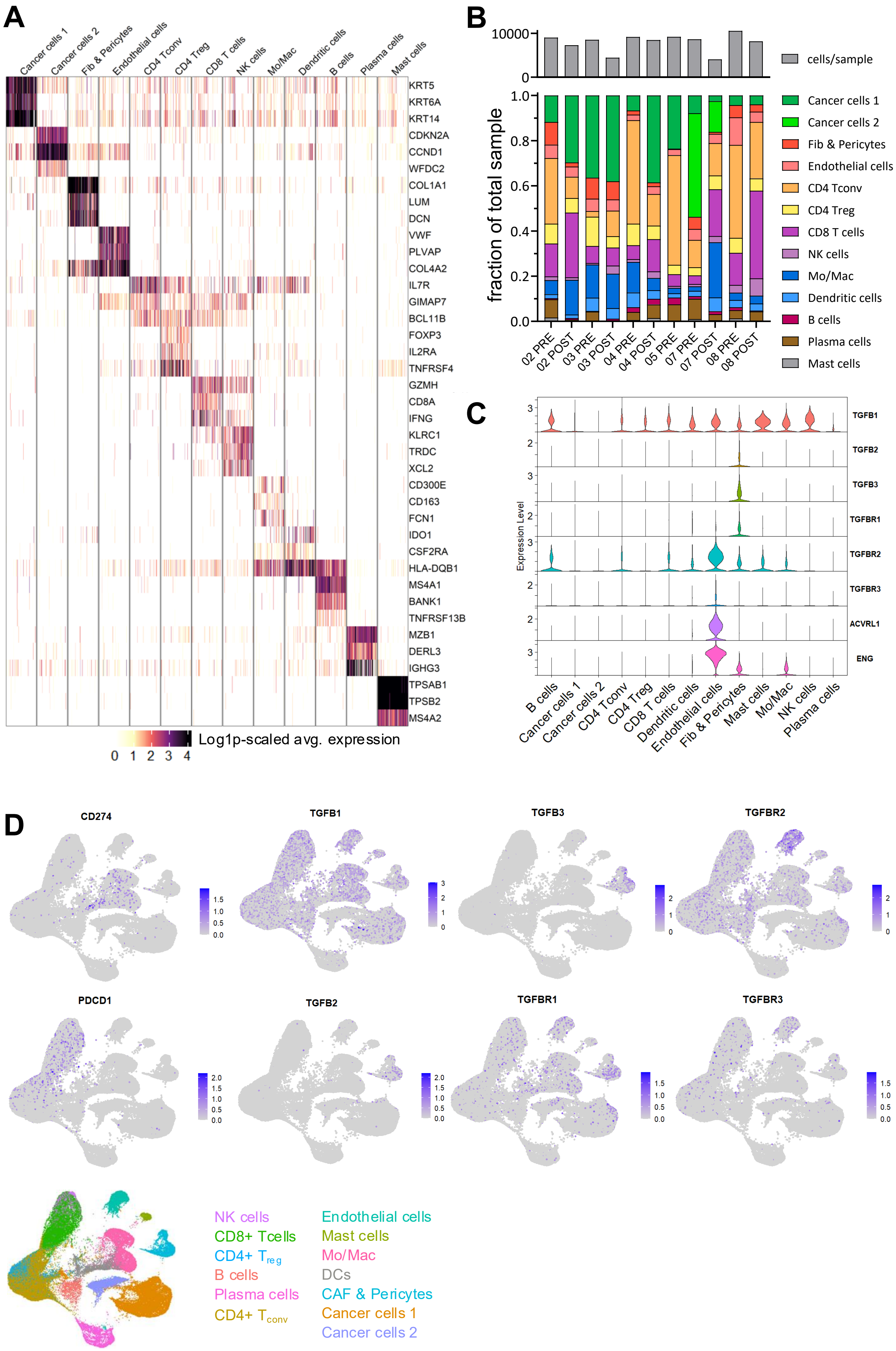
A
Cancer cells 1
Cancer cells 2
Fib & Pericytes
Endothelial cells
CD4 Tconv
CD4 Treg
CD8 T cells
NK cells
Mo/Mac
Dendritic cells
B cells
Plasma cells
Mast cells
KRT5
KRT6A
KRT14
CDKN2A
CCND1
WFDC2
COL1A1
LUM
DCN
VWF
PLVAP
COL4A2
IL7R
GIMAP7
BCL11B
FOXP3
IL2RA
TNFRSF4
GZMH
CD8A
IFNG
KLRC1
TRDC
XCL2
CD300E
CD163
FCN1
IDO1
CSF2RA
HLA-DQB1
MS4A1
BANK1
TNFRSF13B
MZB1
DERL3
IGHG3
TPSAB1
TPSB2
MS4A2
Log1p-scaled avg. expression
0 1 2 3 4
B
10000
0
cells/sample
fraction of total sample
1.0
0.8
0.6
0.4
0.2
0.0
02 PRE
02 POST
03 PRE
03 POST
04 PRE
04 POST
05 PRE
07 PRE
07 POST
08 PRE
08 POST
Cancer cells 1
Cancer cells 2
Fib & Pericytes
Endothelial cells
CD4 Tconv
CD4 Treg
CD8 T cells
NK cells
Mo/Mac
Dendritic cells
B cells
Plasma cells
Mast cells
C
Expression Level
TGFB1
TGFB2
TGFB3
TGFBR1
TGFBR2
TGFBR3
ACVRL1
ENG
B cells
Cancer cells 1
Cancer cells 2
CD4 Tconv
CD4 Treg
CD8 T cells
Dendritic cells
Endothelial cells
Fib & Pericytes
Mast cells
Mo/Mac
NK cells
Plasma cells
D
CD274
TGFB1
TGFB3
TGFBR2
PDCD1
TGFB2
TGFBR1
TGFBR3
NK cells
CD8+ Tcells
CD4+ T_{reg}
B cells
Plasma cells
CD4+ T_{conv}
Endothelial cells
Mast cells
Mo/Mac
DCs
CAF & Pericytes
Cancer cells 1
Cancer cells 2

Supplementary Fig. 2 | A-D. Single-cell RNA sequencing of 87,645 cells from n = 6 PRE and n = 5 POST samples after QC filtering and clustering. **A**, Heatmap showing representative marker genes per main cell types. **B,** Total cell number and abundance of cell types per sample. **C,** Violin plot of TGFβ and main TGFβ receptors expression across cell types. **D,** PD-L1 (CD274), PD1 (PDCD1), TGFβ and main TGFβ receptors projected on UMAP.

Figure S3

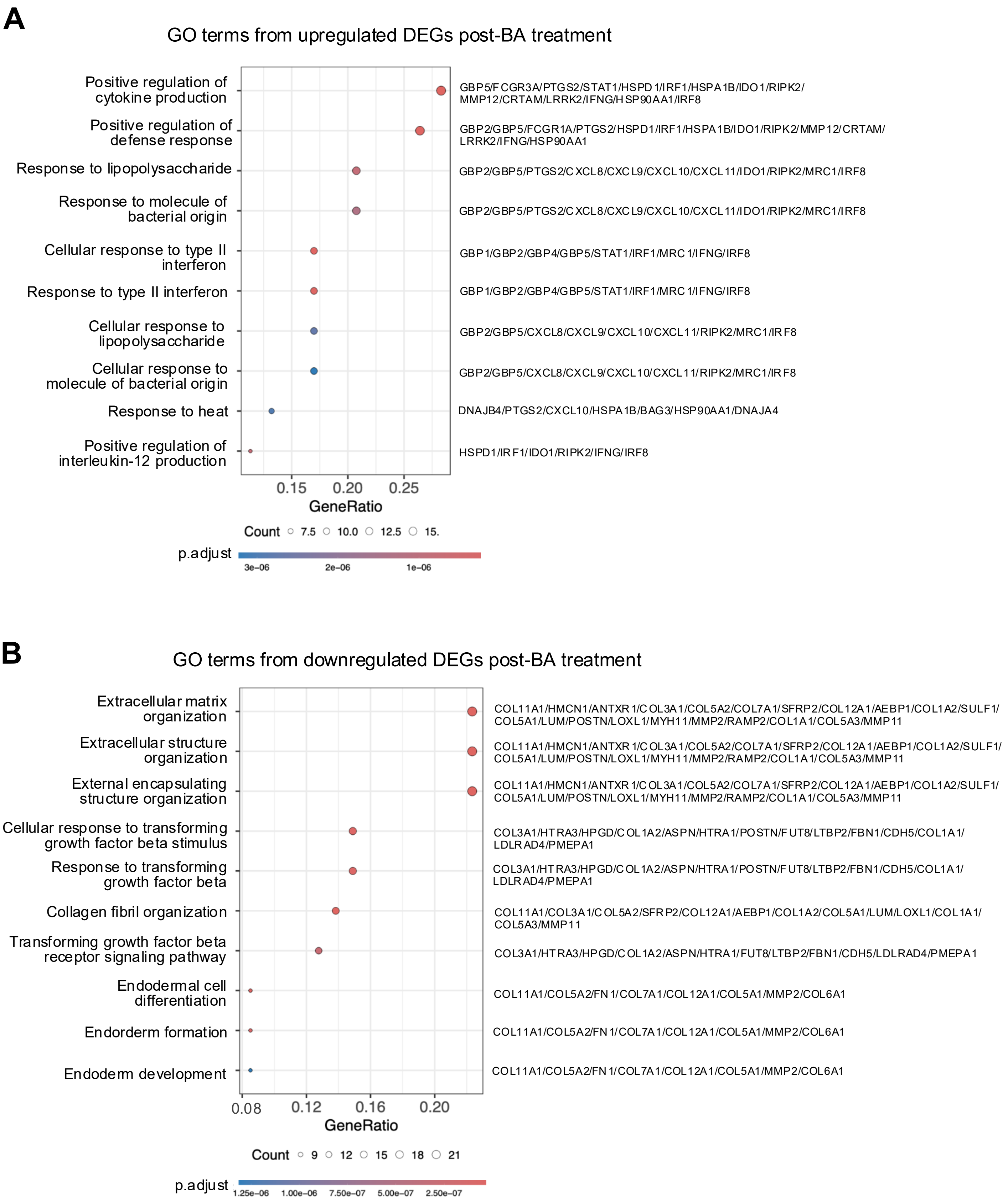
A
GO terms from upregulated DEGs post-BA treatment
Positive regulation of cytokine production
Positive regulation of defense response
Response to lipopolysaccharide
Response to molecule of bacterial origin
Cellular response to type II interferon
Response to type II interferon
Cellular response to lipopolysaccharide
Cellular response to molecule of bacterial origin
Response to heat
Positive regulation of interleukin-12 production
GBP5/FCGR3A/PTGS2/STAT1/HSPD1/IRF1/HSPA1B/IDO1/RIPK2/MMP12/CRTAM/LRRK2/IFNG/HSP90AA1/IRF8
GBP2/GBP5/FCGR1A/PTGS2/HSPD1/IRF1/HSPA1B/IDO1/RIPK2/MMP12/CRTAM/LRRK2/IFNG/HSP90AA1
GBP2/GBP5/PTGS2/CXCL8/CXCL9/CXCL10/CXCL11/IDO1/RIPK2/MRC1/IRF8
GBP2/GBP5/PTGS2/CXCL8/CXCL9/CXCL10/CXCL11/IDO1/RIPK2/MRC1/IRF8
GBP1/GBP2/GBP4/GBP5/STAT1/IRF1/MRC1/IFNG/IRF8
GBP1/GBP2/GBP4/GBP5/STAT1/IRF1/MRC1/IFNG/IRF8
GBP2/GBP5/CXCL8/CXCL9/CXCL10/CXCL11/RIPK2/MRC1/IRF8
GBP2/GBP5/CXCL8/CXCL9/CXCL10/CXCL11/RIPK2/MRC1/IRF8
DNAJB4/PTGS2/CXCL10/HSPA1B/BAG3/HSP90AA1/DNAJA4
HSPD1/IRF1/IDO1/RIPK2/IFNG/IRF8
0.15
0.20
0.25
GeneRatio
Count 7.5 10.0 12.5 15.
p.adjust
3e-06 2e-06 1e-06
B
GO terms from downregulated DEGs post-BA treatment
Extracellular matrix organization
Extracellular structure organization
External encapsulating structure organization
Cellular response to transforming growth factor beta stimulus
Response to transforming growth factor beta
Collagen fibril organization
Transforming growth factor beta receptor signaling pathway
Endodermal cell differentiation
Endorderm formation
Endoderm development
COL11A1/HMCN1/ANTXR1/COL3A1/COL5A2/COL7A1/SFRP2/COL12A1/AEBP1/COL1A2/SULF1/COL5A1/LUM/POSTN/LOXL1/MYH11/MMP2/RAMP2/COL1A1/COL5A3/MMP11
COL11A1/HMCN1/ANTXR1/COL3A1/COL5A2/COL7A1/SFRP2/COL12A1/AEBP1/COL1A2/SULF1/COL5A1/LUM/POSTN/LOXL1/MYH11/MMP2/RAMP2/COL1A1/COL5A3/MMP11
COL11A1/HMCN1/ANTXR1/COL3A1/COL5A2/COL7A1/SFRP2/COL12A1/AEBP1/COL1A2/SULF1/COL5A1/LUM/POSTN/LOXL1/MYH11/MMP2/RAMP2/COL1A1/COL5A3/MMP11
COL3A1/HTRA3/HPGD/COL1A2/ASPN/HTRA1/POSTN/FUT8/LTBP2/FBN1/CDH5/COL1A1/LDLRAD4/PMEPA1
COL3A1/HTRA3/HPGD/COL1A2/ASPN/HTRA1/POSTN/FUT8/LTBP2/FBN1/CDH5/COL1A1/LDLRAD4/PMEPA1
COL11A1/COL3A1/COL5A2/SFRP2/COL12A1/AEBP1/COL1A2/COL5A1/LUM/LOXL1/COL1A1/COL5A3/MMP11
COL3A1/HTRA3/HPGD/COL1A2/ASPN/HTRA1/FUT8/LTBP2/FBN1/CDH5/LDLRAD4/PMEPA1
COL11A1/COL5A2/FN1/COL7A1/COL12A1/COL5A1/MMP2/COL6A1
COL11A1/COL5A2/FN1/COL7A1/COL12A1/COL5A1/MMP2/COL6A1
COL11A1/COL5A2/FN1/COL7A1/COL12A1/COL5A1/MMP2/COL6A1
0.08
0.12
0.16
0.20
GeneRatio
Count 9 12 15 18 21
p.adjust
1.25e-06 1.00e-06 7.50e-07 5.00e-07 2.50e-07

Supplementary Fig. 3 | Gene ontology (GO) enrichment analysis from post-BA treatment DEG analysis. A-B. Dot plots showing the top enriched GO terms (biological process gene sets) in the POST upregulated (A) and downregulated (B) DEGs after over-representation analysis. X axis indicates the number of genes observed in the gene set divided by the total number of genes in the set. Dot gradient color indicates the adjusted p-value. Right panels: list of DEGs associated with the corresponding GO term.

Figure S4

Franken et al. (2024)
HNSCC aPD-L1 (Durvalumab)

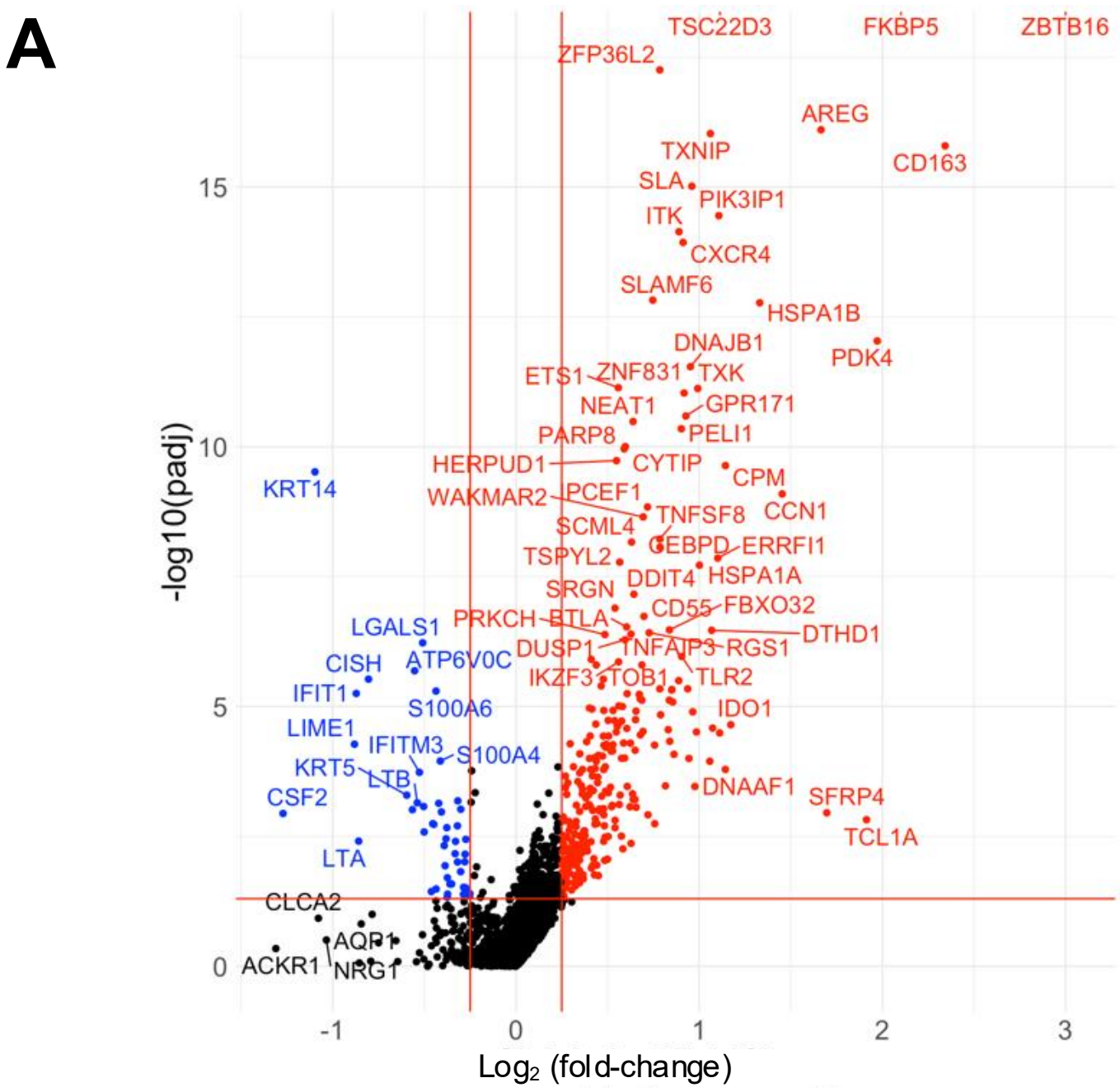

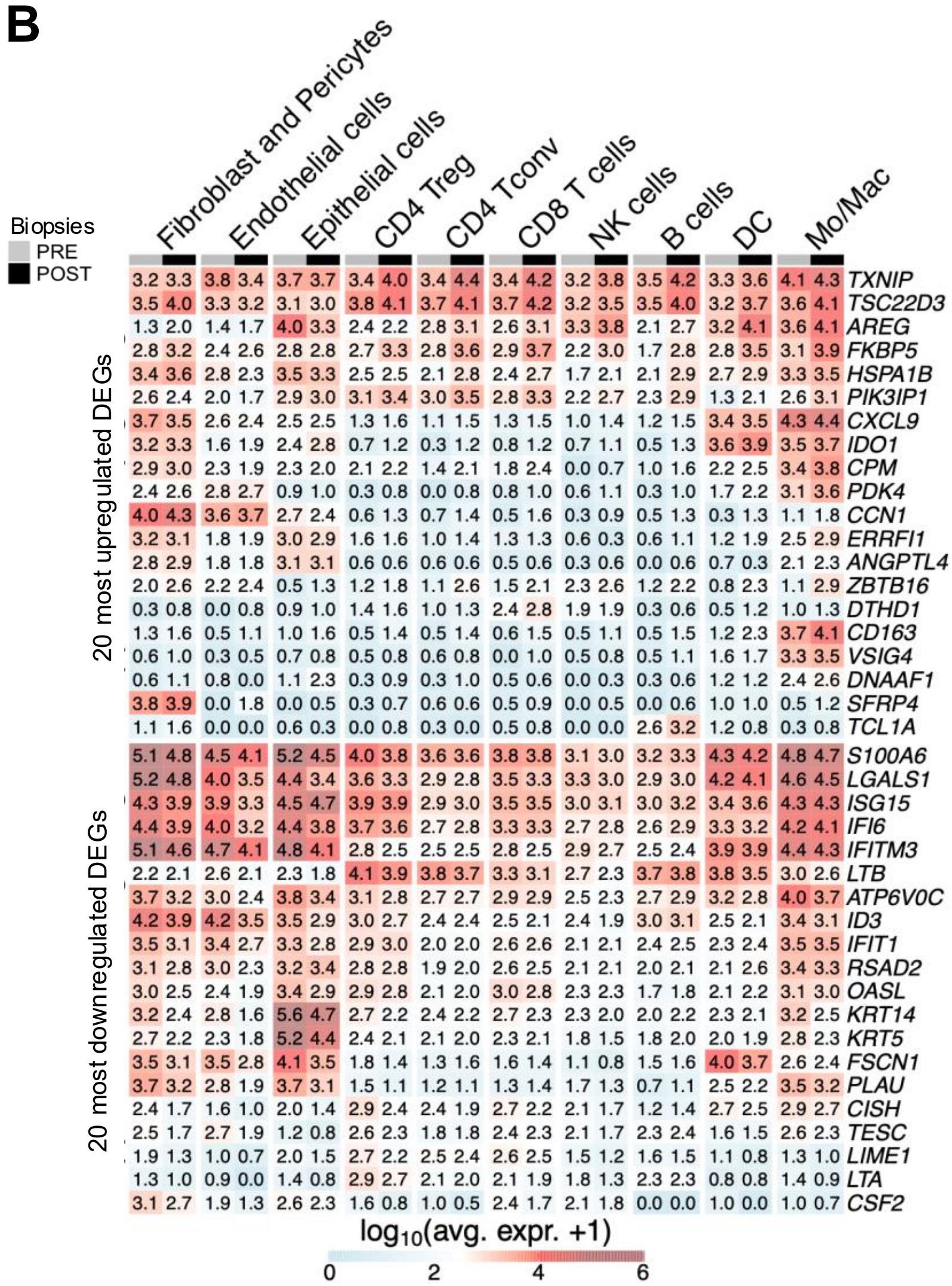

Supplementary Fig. 4 | Analysis of a published scRNAseq dataset of 10 pre and 10 post paired samples from HNSCC patients treated by Durvalumab (aPD-L1) (Franken et al., 2024). A. Volcano plot of the DEGs between pre- and post-treatment samples after pre-/post-treatment, patient, and cluster pseudo-bulking (blue = downregulated, red = upregulated, in post-treatment samples). **B.** Expression level of the top 20 up- and down-regulated DEGs from the analyses in A, shown within main cell types in the paired pre- and post-treatment samples. Heatmap showing the gene expression level of the top 20 up- and down-regulated DEGs from the analysis in A, shown for each cell type in pre- and post-treatment conditions, respectively. Analysis performed with the n=10 paired pre- and post-treatment samples. Values represent log_{10}-transformed average expression levels (a difference of 1 corresponds to a 10-fold difference in expression).

Figure S5

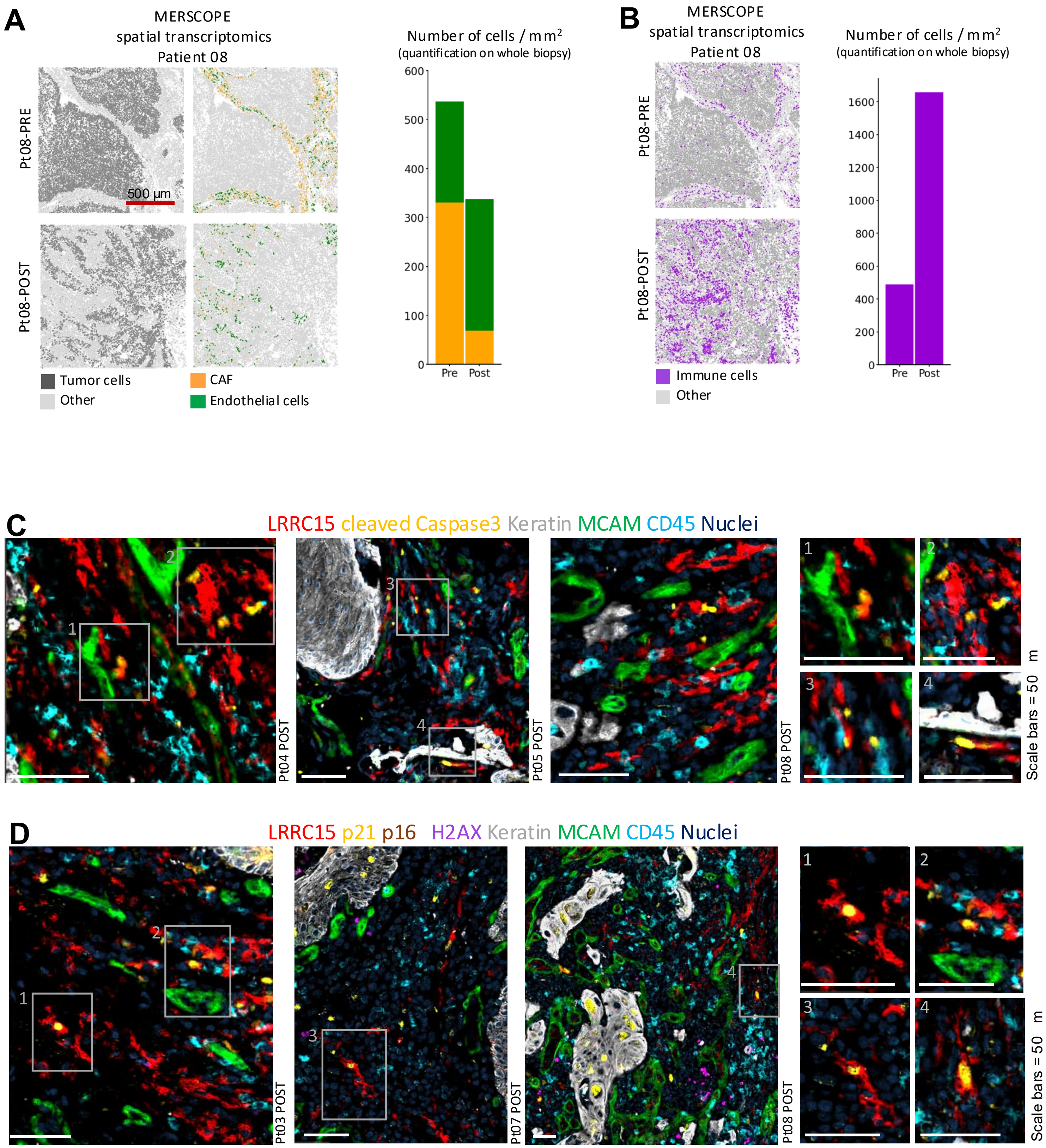
A
MERSCOPE
spatial transcriptomics
Patient 08
Pt08-PRE
Pt08-POST
500 µm
Tumor cells
Other
CAF
Endothelial cells
Number of cells / mm²
(quantification on whole biopsy)
0
100
200
300
400
500
600
Pre
Post
B
MERSCOPE
spatial transcriptomics
Patient 08
Pt08-PRE
Pt08-POST
Immune cells
Other
Number of cells / mm²
(quantification on whole biopsy)
0
200
400
600
800
1000
1200
1400
1600
Pre
Post
C
LRRC15 cleaved Caspase3 Keratin MCAM CD45 Nuclei
Pt04 POST
Pt05 POST
Pt08 POST
Scale bars = 50 m
D
LRRC15 p21 p16 H2AX Keratin MCAM CD45 Nuclei
Pt03 POST
Pt07 POST
Pt08 POST
Scale bars = 50 m

Supplementary Fig. 5 | A-B, MERFISH spatial transcriptomics (n=1, Pt08). **A,** Tumor and stromal cell detection (left) and cell density quantification (right). **B,** Immune cell detection (left) and cell density quantification (right). **C-D,** Examples of multiplex IHC images of apoptosis (cleaved caspase 3, **(C)**) and senescence (p21, p16, γH2AX **(D)**) markers in POST biopsies. Right panels: higher magnification images. Scale bars: 50 μm.

Figure S6

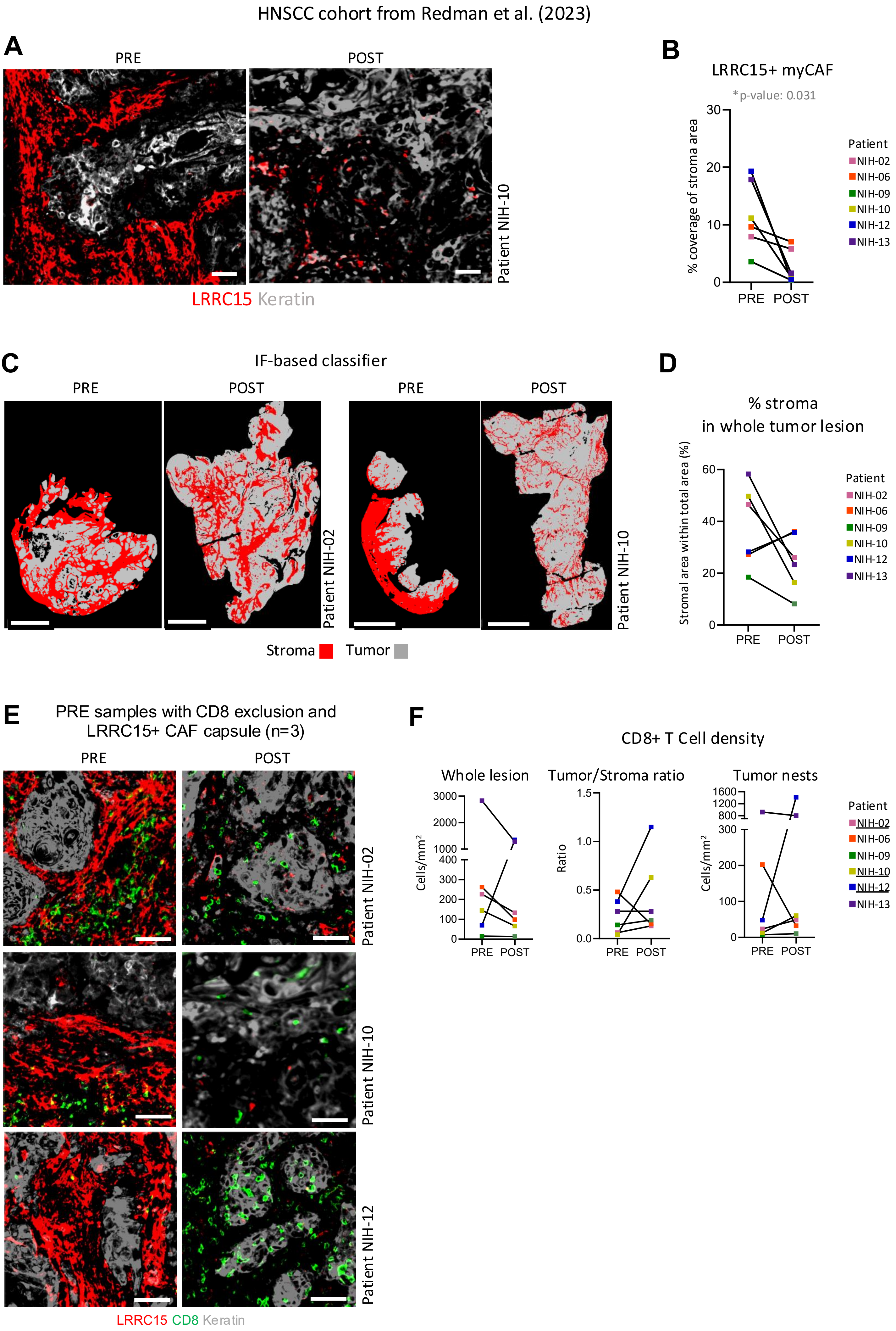

HNSCC cohort from Redman et al. (2023)
A
PRE
POST
Patient NIH-10
LRRC15 Keratin
B
LRRC15+ myCAF
*p-value: 0.031
% coverage of stroma area
PRE
POST
Patient
NIH-02
NIH-06
NIH-09
NIH-10
NIH-12
NIH-13
C
IF-based classifier
PRE
POST
Patient NIH-02
Patient NIH-10
Stroma
Tumor
D
% stroma
in whole tumor lesion
Stromal area within total area (%)
E
PRE samples with CD8 exclusion and
LRRC15+ CAF capsule (n=3)
Patient NIH-12
LRRC15 CD8 Keratin
F
CD8+ T Cell density
Whole lesion
Tumor/Stroma ratio
Tumor nests
Cells/mm²
Ratio

Supplementary Fig. 6 | Validation of stromal and CD8+ T cell infiltration changes in an independent BA-treated cohort of 6 HNSCC patients (Redman et al, 2022). A, Representative OPAL image of PRE and POST biopsies stained for LRRC15 and pan-Keratin. Scale bars = 50 µm. **B,** Stromal area coverage of LRRC15+ myCAF as quantified for each biopsy. Wilcoxon paired test, p-value = 0.031. **C,** Example of tumor and stroma classifier (QuPath analysis) on PRE and POST biopsies for two patients with pronounced decrease in the stromal compartment following BA treatment (NIH-Pts 02 and 10). **D,** Quantification of the area covered by the stromal compartment within the whole tumor lesion for all paired samples. Wilcoxon paired test, p-value = 0.156. **E,** OPAL images from NIH-Pts 02; 10; 12 presenting an LRRC15+ CAF capsule architecture and CD8+ T cell exclusion prior to BA treatment. Right panel: POST-treatment images showing loss of LRRC15+ CAF and increase in CD8+ intratumoral infiltration. Scale bars = 50µm. **F,** Quantification of CD8+ T cell infiltration. Wilcoxon paired test, p-values for whole lesion density = 0.313; for tumor-stroma ratio = 0.313; for density in tumor nests = 0.844.

Figure S7

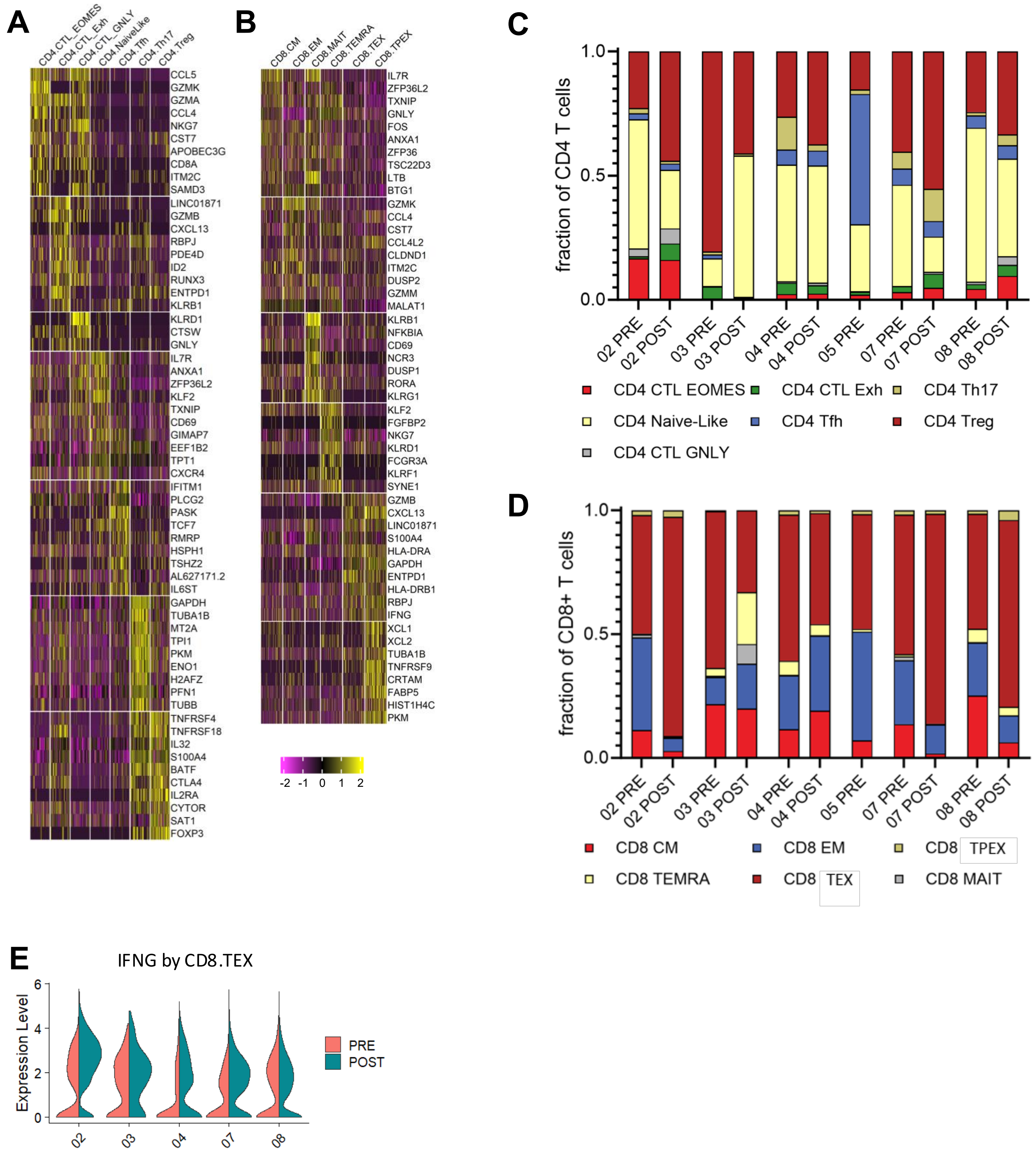
A
CD4.CTL_EOMES
CD4.CTL_Exh
CD4.CTL_GNLY
CD4.NaiveLike
CD4.Tfh
CD4.Th17
CD4.Treg
CCL5 GZMK GZMA CCL4 NKG7 CST7 APOBEC3G CD8A ITM2C SAMD3
LINC01871 GZMB CXCL13 RBPJ PDE4D ID2 RUNX3 ENTPD1 KLRB1
KLRD1 CTSW GNLY
IL7R ANXA1 ZFP36L2 KLF2 TXNIP CD69 GIMAP7 EEF1B2 TPT1 CXCR4
IFITM1 PLCG2 PASK TCF7 RMRP HSPH1 TSHZ2 AL627171.2 IL6ST
GAPDH TUBA1B MT2A TPI1 PKM ENO1 H2AFZ PFN1 TUBB
TNFRSF4 TNFRSF18 IL32 S100A4 BATF CTLA4 IL2RA CYTOR SAT1 FOXP3
B
CD8.CM
CD8.EM
CD8.MAIT
CD8.TEMRA
CD8.TEX
CD8.TPEX
IL7R ZFP36L2 TXNIP GNLY FOS ANXA1 ZFP36 TSC22D3 LTB BTG1
GZMK CCL4 CST7 CCL4L2 CLDND1 ITM2C DUSP2 GZMM MALAT1
KLRB1 NFKBIA CD69 NCR3 DUSP1 RORA KLRG1
KLF2 FGFBP2 NKG7 KLRD1 FCGR3A KLRF1 SYNE1
GZMB CXCL13 LINC01871 S100A4 HLA-DRA GAPDH ENTPD1 HLA-DRB1 RBPJ IFNG
XCL1 XCL2 TUBA1B TNFRSF9 CRTAM FABP5 HIST1H4C PKM
-2 -1 0 1 2
C
fraction of CD4 T cells
1.0
0.5
0.0
02 PRE
02 POST
03 PRE
03 POST
04 PRE
04 POST
05 PRE
07 PRE
07 POST
08 PRE
08 POST
CD4 CTL EOMES
CD4 CTL Exh
CD4 Th17
CD4 Naive-Like
CD4 Tfh
CD4 Treg
CD4 CTL GNLY
D
fraction of CD8+ T cells
1.0
0.5
0.0
02 PRE
02 POST
03 PRE
03 POST
04 PRE
04 POST
05 PRE
07 PRE
07 POST
08 PRE
08 POST
CD8 CM
CD8 EM
CD8 TPEX
CD8 TEMRA
CD8 TEX
CD8 MAIT
E
IFNG by CD8.TEX
Expression Level
6
4
2
0
02
03
04
07
08
PRE
POST

Supplementary Fig. 7 | ScRNAseq analysis of T cell subsets. A-B. Heatmap of top 10 deduplicated identifying genes for (A) CD4+ T cell subsets and (B) CD8+ T cell subsets. **C-D**. Proportional changes of the identified (C) CD4+ T cell subsets and (D) CD8+ T cell subsets within CD4+ T cells. **E.** IFNG expression by CD8.TEX in each patient.

Figure S8

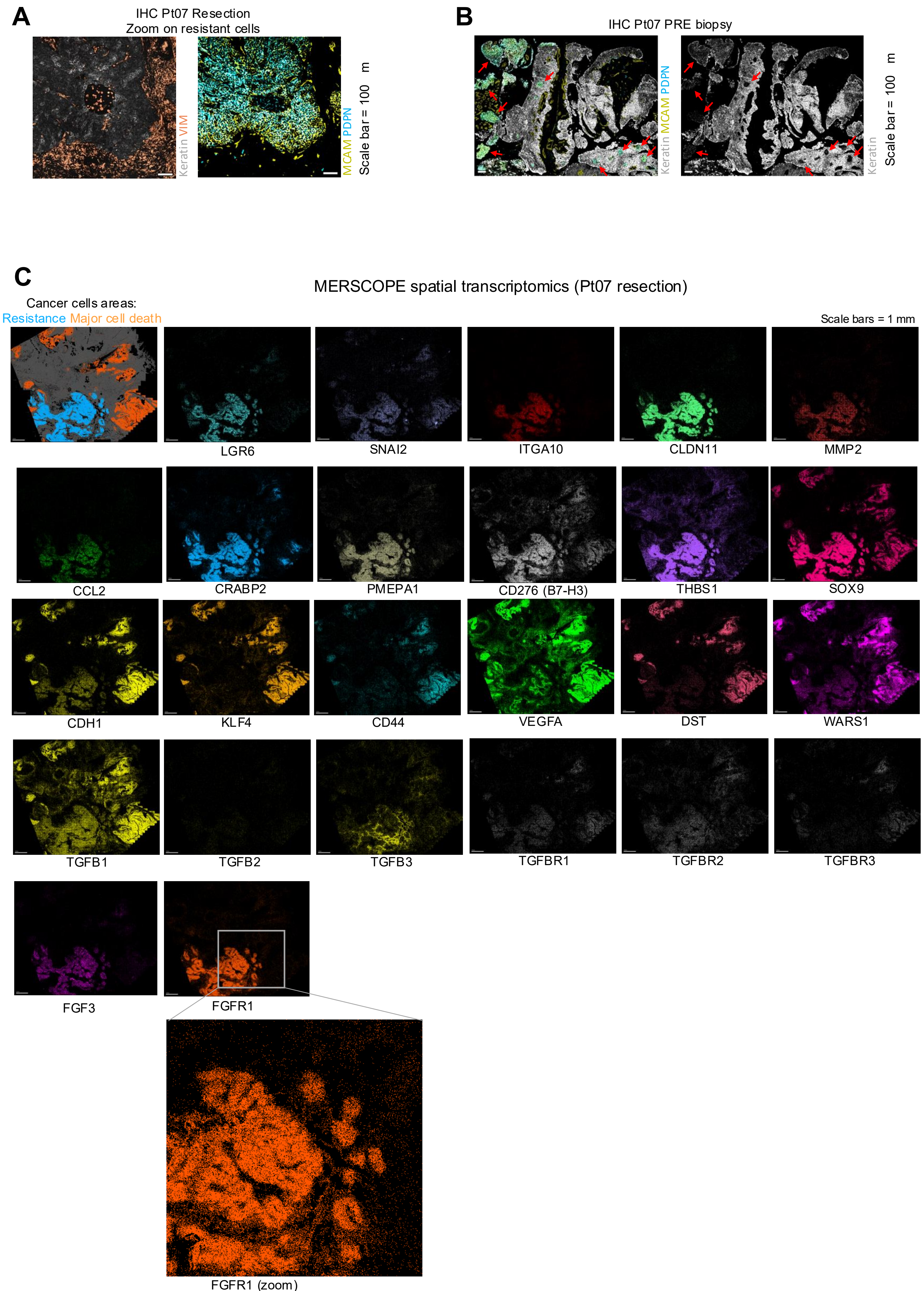
A
IHC Pt07 Resection
Zoom on resistant cells
Keratin VIM
MCAM PDPN
Scale bar = 100 m
B
IHC Pt07 PRE biopsy
Keratin MCAM PDPN
Keratin
Scale bar = 100 m
C
MERSCOPE spatial transcriptomics (Pt07 resection)
Cancer cells areas:
Resistance Major cell death
Scale bars = 1 mm
LGR6
SNAI2
ITGA10
CLDN11
MMP2
CCL2
CRABP2
PMEPA1
CD276 (B7-H3)
THBS1
SOX9
CDH1
KLF4
CD44
VEGFA
DST
WARS1
TGFB1
TGFB2
TGFB3
TGFBR1
TGFBR2
TGFBR3
FGF3
FGFR1
FGFR1 (zoom)

Supplementary Fig. 8 | Differences between resistant and sensitive cancer cells. A. Left: multiplex IF staining (Orion) of pan-Keratin and Vimentin of the resistant zone shown in Fig. 5A, Right: multiplex IHC staining (MICSSS) of MCAM and PDPN of the same area. **B.** MICSSS image of the MPR's pre-treatment biopsy containing cancer cells with the same staining profile as the resistant cancer cells in the resection, highlighted by red arrows. **C.** MERFISH data visualization for single genes. Each colored pixel corresponds to one detected transcript.
